# Traumatic brain injury stimulates sympathetic tone-mediated bone marrow myelopoiesis to favor fracture healing

**DOI:** 10.1038/s41392-023-01457-w

**Published:** 2023-07-05

**Authors:** Weijian Liu, Wei Chen, Mao Xie, Chao Chen, Zengwu Shao, Yiran Zhang, Haiyue Zhao, Qingcheng Song, Hongzhi Hu, Xin Xing, Xianyi Cai, Xiangtian Deng, Xinyan Li, Peng Wang, Guohui Liu, Liming Xiong, Xiao Lv, Yingze Zhang

**Affiliations:** 1 Department of Orthopaedics, Union Hospital, Tongji Medical College, Huazhong University of Science and Technology, Wuhan, 430022 China; 2 Department of Orthopaedic Surgery, The Third Hospital of Hebei Medical University, Shijiazhuang, 050051 China; 3 School of Medicine, Nankai University, Tianjin, 300071 China; 4 NHC Key Laboratory of Intelligent Orthopeadic Equipment, Third Hospital of Hebei Medical University, Shijiazhuang, 050051 China; 5 Animal Center of Hebei Ex & In vivo Biotechnology, Shijiazhuang, 050051 China; 6 Department of Physiology, School of Basic Medicine and Tongji Medical College, Huazhong University of Science and Technology, Wuhan, 430030 China

**Keywords:** Rheumatic diseases, Trauma

## Abstract

Traumatic brain injury (TBI) accelerates fracture healing, but the underlying mechanism remains largely unknown. Accumulating evidence indicates that the central nervous system (CNS) plays a pivotal role in regulating immune system and skeletal homeostasis. However, the impact of CNS injury on hematopoiesis commitment was overlooked. Here, we found that the dramatically elevated sympathetic tone accompanied with TBI-accelerated fracture healing; chemical sympathectomy blocks TBI-induced fracture healing. TBI-induced hypersensitivity of adrenergic signaling promotes the proliferation of bone marrow hematopoietic stem cells (HSCs) and swiftly skews HSCs toward anti-inflammation myeloid cells within 14 days, which favor fracture healing. Knockout of β3- or β2-adrenergic receptor (AR) eliminate TBI-mediated anti-inflammation macrophage expansion and TBI-accelerated fracture healing. RNA sequencing of bone marrow cells revealed that Adrb2 and Adrb3 maintain proliferation and commitment of immune cells. Importantly, flow cytometry confirmed that deletion of β2-AR inhibits M2 polarization of macrophages at 7th day and 14th day; and TBI-induced HSCs proliferation was impaired in β3-AR knockout mice. Moreover, β3- and β2-AR agonists synergistically promote infiltration of M2 macrophages in callus and accelerate bone healing process. Thus, we conclude that TBI accelerates bone formation during early stage of fracture healing process by shaping the anti-inflammation environment in the bone marrow. These results implicate that the adrenergic signals could serve as potential targets for fracture management.

## Introduction

Fractures combined with traumatic brain injury (TBI) are commonly observed in industrialized society.^1,2^ Generally, patients suffered from TBI shows an accelerate fracture healing rate and callus ossification.^3,4^ The role of circulating growth factors, alkalotic environment, noncoding RNAs and small extracellular vesicles have been established in TBI-accelerated fracture healing.^5–10^ However, whether there is a direct association between the central nervous system (CNS) and accelerated bone healing followed with TBI remains unclear.

Bone marrow provides a unique microenvironment for hematopoietic stem cells (HSCs) and bone marrow mesenchymal stem cells (bMSCs).^11,12^ The skeleton homeostasis was maintained by continuous bone remodeling, which is accomplished by HSCs lineage osteoclasts-induced bone resorption and bMSCs-derived osteoblasts-mediated bone formation. Likewise, HSCs mediate the lifelong production of blood cells, and immune cells include lymphocytes and myeloid cells to maintain the homeostasis of the immune system.^13^ HSCs reside in bone marrow niche in a quiescent state and is regulated by bMSCs.^14^ Inflammation, medicine intake and trauma activate stem cells for emergency hematopoiesis, and trigger the expansion of multiple HSCs lineage cells.^15^ During the initial stage of fracture, multiple immune cells are produced by HSCs, and migrate into the fracture gap, leading to the hematoma and callus formation.^16^ Importantly, myeloid cells, which promote mineralization and angiogenesis in the callus, contribute to the bone healing process.^17–20^ Moreover, deletion of macrophage abrogated osteogenesis and callus formation, indicating an essential role of macrophages in fracture healing.^21–23^ We previously reported that 5–10% fractures concurrent complaint such as delayed union or non-union, which impair physiological function and psychosocial well-being.^24,25^ Therefore, a better understanding of the relationship between TBI-induced myelopoiesis and fracture healing will provide a promising strategy for accelerating fracture healing and treating fracture non-union.

The pivotal role of the CNS in regulating peripheral organs was established for decades.^26^ Long-range interactions between CNS and immune system permit the nervous system to regulate immune cells in bone marrow.^27^ Notably, sympathetic nerves penetrate the bone marrow tightly and precisely regulate skeletal and hematopoietic homeostasis.^11,28^ We have demonstrated that skeletal interoception maintains bone homeostasis and differentiation of bMSCs by regulating sympathetic nervous system.^29–31^ The sympathetic nerves orchestrate the mobilization and egress of HSCs through adrenergic signaling in the stem cell niche.^32,33^ Briefly, myeloid expansion during hematopoiesis relies on the β2-adrenergic receptors (ARs).^14,34,35^ Importantly, β2-ARs are essential for tissue regeneration after trauma or acute injury.^36,37^ β3-ARs maintain the HSCs’ proliferation, self-maintenance, as well as the lymphoid skewing.^14,38^ Activating of β3-ARs rejuvenated the function of aged HSCs.^14^ As patients with severe TBI often exhibited a long-lasting sympathetic nervous system (SNS) overactivity,^39,40^ we hypothesize that TBI promotes fracture healing through activating myeloid hematopoiesis by tuning SNS.

In this study, we demonstrate that TBI-mediated activation of SNS accelerates fracture healing process. SNS-expanded hematopoiesis and myeloid bias are essential for this beneficial effect. In addition, knockout of β3- and β2-ARs eliminates TBI-mediated myelopoiesis and expansion of anti-inflammation myeloid cells. In agreement with these findings, selective activation of β2- and β3-ARs synergistically promote bone healing process. We conclude that activated SNS drives myelopoiesis to facilitate fracture healing in mice with TBI.

## Results

### Sympathetic tones in bone marrow elevated after TBI

**Fig. 1:**
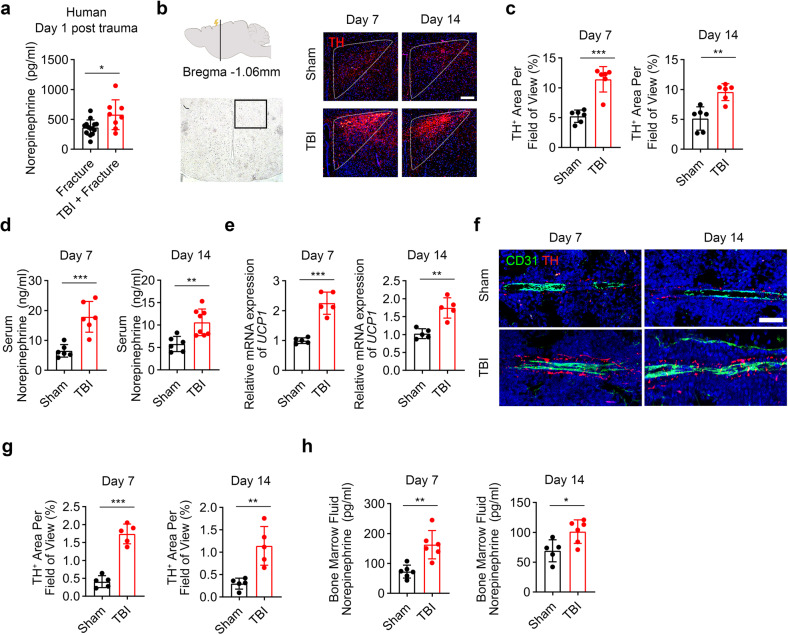
TBI increases the sympathetic tone. **a** Quantitative analysis of serum norepinephrine (NE) by ELISA assay from fracture patients with or without concomitant TBI. **b**, **c** Representative immunofluorescence (IF) staining and quantitative analysis of TH expression in the paraventricular nucleus (PVN) of hypothalamus from 4-month-old male mice with fracture combined sham or TBI operation at the 7th day and 14th day post operation. Scale bar: 200 μm. **d** Quantitative analysis of serum NE by ELISA assay from 4-month-old male mice with fracture combined sham or TBI operation at the 7th day and 14th day post operation. **e** Quantitative analysis of mRNA expression of UCP1 in brown adipose tissue from 4-month-old male mice with fracture combined sham or TBI operation at the 7th day and 14th day post operation. **f**, **g** Representative IF staining and quantitative analysis of TH expression in the bone marrow from 4-month-old male mice with fracture combined sham or TBI operation at 7th day and 14th day post operation. Scale bar: 50 μm. **h** Quantitative analysis of bone marrow fluid NE by ELISA assay from 4-month-old male mice with fracture combined sham or TBI operation at the 7th day and 14th day post operation. All data are presented as means ± standard error of the mean (SEM). *P < 0.05, **P < 0.01 and ***P < 0.001. Statistical significance was determined by two-tailed Student’s *t* test

Accelerated fracture healing rates were observed in patients suffered from femur fracture subjected to TBI (Supplementary Fig. 1). To test whether TBI affects the sympathetic tone in concomitant fracture, the levels of serum norepinephrine (NE) were measured in patients suffered from femur fracture subjected with TBI (Supplementary Tables 1 and 2). TBI significantly stimulated the secretion of NE (Fig. 1a). We prepared femur-fractured mice combined with sham or TBI operation through weight-drop device (Supplementary Fig. 2).^41^ Hypothalamic paraventricular nucleus (PVN) is known critical for sympathetic outflow.^42^ Immunostaining showed significantly elevated expression of tyrosine hydroxylase (TH) in PVN areas in TBI-treated mice relative to the sham group (Fig. 1b, c). The NE levels in serum elevated significantly in the TBI group compared with sham group both at 7th day and 14th day post TBI (Fig. 1d). Uncoupling protein 1 gene (UCP1) expression in interscapular brown adipose tissue increased significantly at the 7th day and 14th day post fracture in the TBI-treated mice relative to the sham group (Fig. 1e). These results indicate higher sympathetic tone in this brain injury model. Sympathetic nerves regulate bone marrow cells via the adrenergic signaling. Immunostaining showed significantly increased TH+ sympathetic noradrenergic fibers in bone marrow in TBI group (Fig. 1f, g). In addition, the NE levels in bone marrow supernatant elevated significantly in TBI group compared with the sham group both at 7th day and 14th day post TBI (Fig. 1h). Together, these results suggest that the sympathetic tones in bone marrow were activated after TBI.

### Sympathetic nerves are essential for TBI-accelerated fracture healing through increasing the infiltration of macrophages and type H vessels

**Fig. 2:**
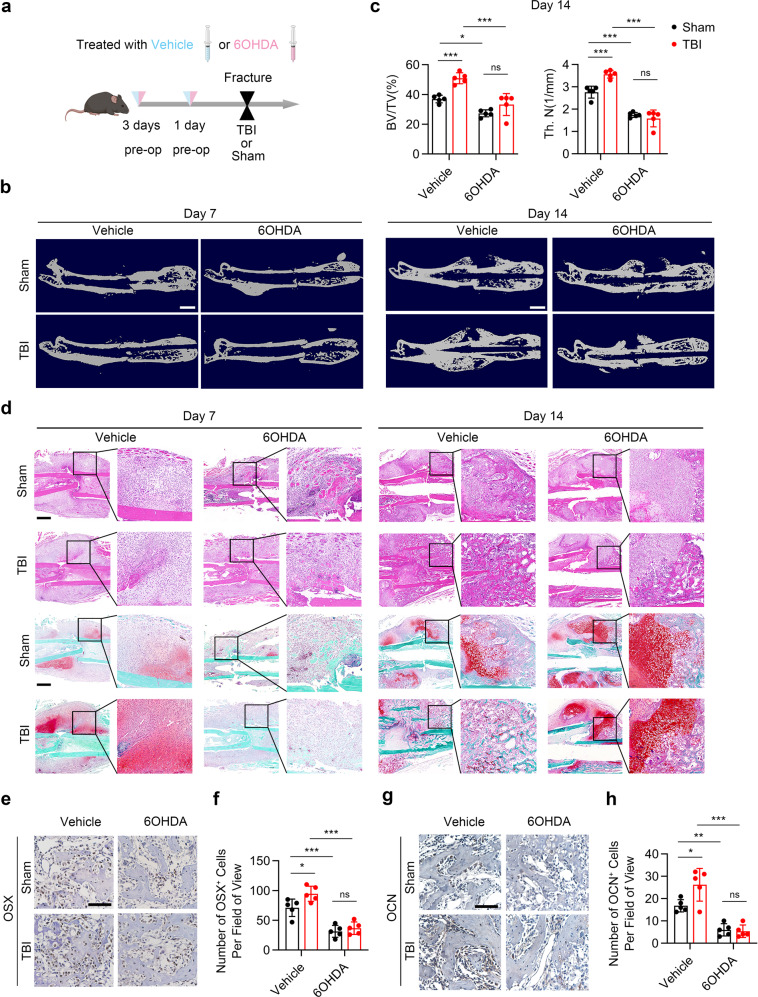
TBI promotes fracture healing through sympathetic nerve. **a** Schematic graph of the study of TBI model and 6-OHDA injection procedure. Male C57B6/J mice were treated with vehicle or 6-OHDA at a dose at 100 mg/kg by intraperitoneal injection 3 days before operation and a dose at 250 mg/kg 1 day before operation; the fractured mice were treated with sham operation or TBI operation. **b** Representative *μ*CT images of fractured femurs from mice at the 7th day and 14th day post operation. Scale bar: 2 mm. **c** Quantitative analysis of bone volume fraction (BV/TV) and trabecular number (Tb.N). **d** Representative HE and SO/FG staining images in the callus area in 4-month-old male mice. Scale bar: 800 μm. **e**, **f** Representative immunohistochemical (IHC) staining and quantitative analysis of Osterix expression in the callus from 4-month-old male mice at 14th day post operation. Scale bar: 100 μm. **g**, **h** Representative IHC staining and quantitative analysis of OCN expression in the callus from 4-month-old male mice at 14th day post operation. Scale bar: 100 μm. All data are presented as means ± standard error of the mean (SEM). *P < 0.05, **P < 0.01, and ***P < 0.001, ns: not significant. Statistical significance was determined by two-way ANOVA

To examine whether TBI promotes fracture healing by tuning sympathetic tones, the mice from the TBI group were treated with 6-hydoxydopamine (6-OHDA) before femur fracture operation (Fig. 2a).^14^ The traumatic injury of the brain was confirmed by a general view of brain tissue (Supplementary Fig. 2). The effect of chemical sympathectomy was tested by immunostaining of TH in the bone marrow and by the NE levels in bone marrow supernatant (Supplementary Fig. 3a,b). As shown by micro-CT (*μ*CT), the bone volume fraction (BV/TV) and the trabecular number (Tb.N) significantly increased in TBI group relative to sham group. Administration of 6-OHDA impaired TBI-accelerated fracture healing process (Fig. 2b, c). Accelerated cartilage callus formation and prominent initiation of mineralization were observed in TBI group at the 7th day and 14th day post fracture, respectively, as showed by H&E staining and safranin O-fast green (SO/FG) staining. The effect of TBI on increased fracture healing rate was abolished in 6-OHDA-treated mice (Fig. 2d). Importantly, the population of CD45^–^CD31^–^Sca-1^+^CD24^+^ MSCs in callus significantly increased in TBI-treated mice at the 14th day post fracture as evidenced through flow cytometry-based measurements.^31,43^ Administrating with 6-OHDA decreased the TBI-induced expansion of MSCs in the callus (Supplementary Fig. 4). Immunohistochemical (IHC) analysis showed that expression of osteocalcin (OCN) and osterix (OSX) in callus areas significantly increased in TBI-treated mice compared with the sham group; whereas chemical sympathectomy arrested these TBI-accelerated fracture healing process (Fig. 2e–h).

**Fig. 3:**
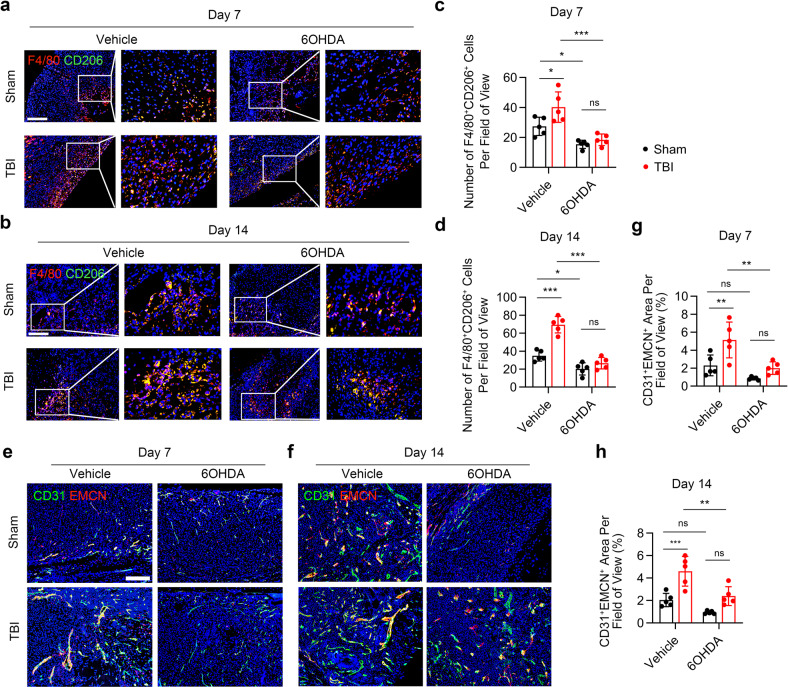
TBI favors M2 macrophages and type H vessels infiltration through sympathetic nerve. **a**–**d** Representative IF staining images and quantitative analysis of M2 macrophages in the callus area in 4-month-old male mice. Scale bar: 200 μm. **e**–**h** Representative IF staining images and quantitative analysis of type H vessels in the callus area in 4-month-old male mice. Scale bar: 200 μm. All data are presented as means ± standard error of the mean (SEM). *P < 0.05, **P < 0.01, and ***P < 0.001, ns: not significant. Statistical significance was determined by two-way ANOVA

The infiltration of alternative activated macrophages, which favor angiogenesis and fracture healing, was examined by immunostaining of F4/80 and CD206 in callus. The results showed significantly elevated number of F4/80^+^CD206^+^ cells in TBI-treat mice compared with the sham group; injection of 6-OHDA reduced the TBI-increased infiltration of F4/80^+^CD206^+^ cells in callus (Fig. 3a–d). Immunostaining of callus displayed significantly increased number of type H vessels in the TBI-treat mice compared with that of the sham group (Fig. 3e–h). However, the TBI-induced angiogenesis was abrogated in mice injected with 6-OHDA (Fig. 3e–h). Together, these results indicate that sympathetic nerves are important for TBI-accelerated fracturing healing through increasing the infiltration of macrophages and type H vessels.

### Sympathetic nerves are essential for TBI-induced bone marrow myelopoiesis

**Fig. 4:**
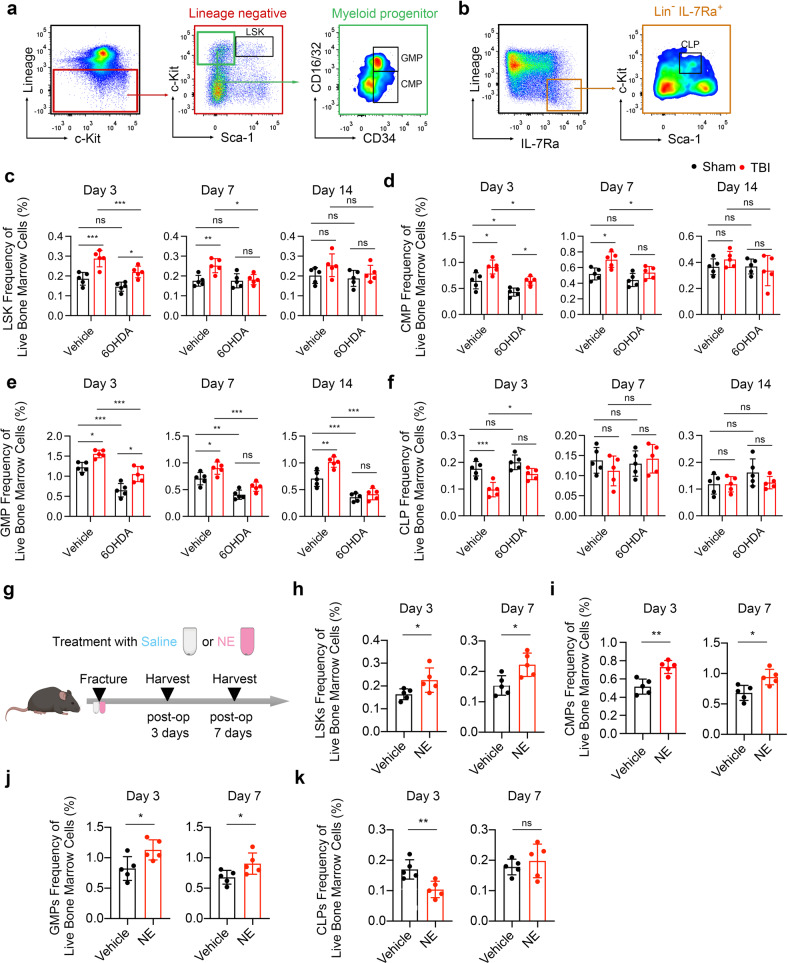
Sympathetic nerve is essential in the bone marrow emergency myelopoiesis after TBI. **a**, **b** Flow cytometry plots show gating strategy for LSKs (Lin^−^ Sca-1^+^ c-Kit^+^ cells), CMPs (Lin^−^ Sca-1^−^ c-Kit^+^ CD34^+^ CD16/32^int^ cells), GMPs (Lin^−^ Sca-1^−^ c-Kit^+^ CD34^+^ CD16/32^+^ cells), and CLPs (Lin^−^ Sca-1^int^ c-Kit^int^ IL7R^+^ cells). **c**–**f** Quantitative analysis of the number of LSKs, CMPs, GMPs, and CLPs isolated from 4-month-old male fractured mice in sham group, 6-OHDA group, TBI group, and TBI + 6-OHDA group. **g** Schematic graph of the study of NE administration procedure. Fractured male C57B6/J mice were treated with vehicle or NE through subcutaneously implanted Alzet osmotic pumps. **h**–**k** Quantitative analysis of the number of LSKs, CMPs, GMPs, and CLPs isolated from 4-month-old male mice treated with vehicle or NE. All data are presented as means ± standard error of the mean (SEM). *P < 0.05, **P < 0.01, and ***P < 0.001, ns: not significant. Statistical significance was determined by two-tailed Student’s *t* test (**h**–**k**) or two-way ANOVA (**c**–**f**)

To investigate whether TBI increases myelopoiesis through sympathetic nerves, we examined the HSC lineage cells in TBI-treated mice administrated with 6-OHDA through flow cytometry-based measurements (Fig. 4a, b). Hematopoietic progenitor cells are defined as lineage^−^ Sca-1^+^ c-Kit^+^ cells (LSKs) that containing multipotent progenitor cells (MPPs), short-term HSCs, and long-term HSCs.^44^ Significantly increased numbers of LSKs were observed in TBI group relative to that of the sham group at the 3rd day and 7th day post fracture (Fig. 4c and Supplementary Fig. 5a). Moreover, the number of myeloid progenitors, including granulocyte monocyte progenitors (GMPs) and common myeloid progenitors (CMPs), significantly increased in TBI group compared with sham group (Fig. 4d, e and Supplementary Fig. 5b). By contrast, the counts of common lymphoid progenitors (CLPs) significantly reduced at the 3rd day after TBI (Fig. 4f and Supplementary Fig. 5c). Importantly, chemical sympathetic excision abrogated the TBI-induced increases of LSKs, CMPs, and GMPs; administration of 6-OHDA also diminished the TBI-mediated decline of CLPs (Fig. 4c–f and Supplementary Fig. 5a–c).

These results drove us to examine the role of β-ARs in the emergency myelopoiesis after fracture. NE is a non-selective β-AR agonist. We examined whether consistent administration of NE simulates the myelopoiesis droved by TBI-elevated sympathetic tone. Mice were administrated with NE or vehicle with Alzet osmotic pumps, and were simultaneously performed with bone fracture model (Fig. 4g). Indeed, the numbers of LSKs, CMPs and GMPs significantly increased in NE-administrated mice at the 3rd day and 7th day post fracture (Fig. 4h–j and Supplementary Fig. 6a, b). Accordingly, the counts of CLPs significantly declined in NE-treated mice only at the 3rd day compared to the vehicle group (Fig. 4k and Supplementary Fig. 6c). These results reveal that sympathetic nerves contribute to TBI-promoted myeloid bias of hematopoiesis.

**Fig. 5:**
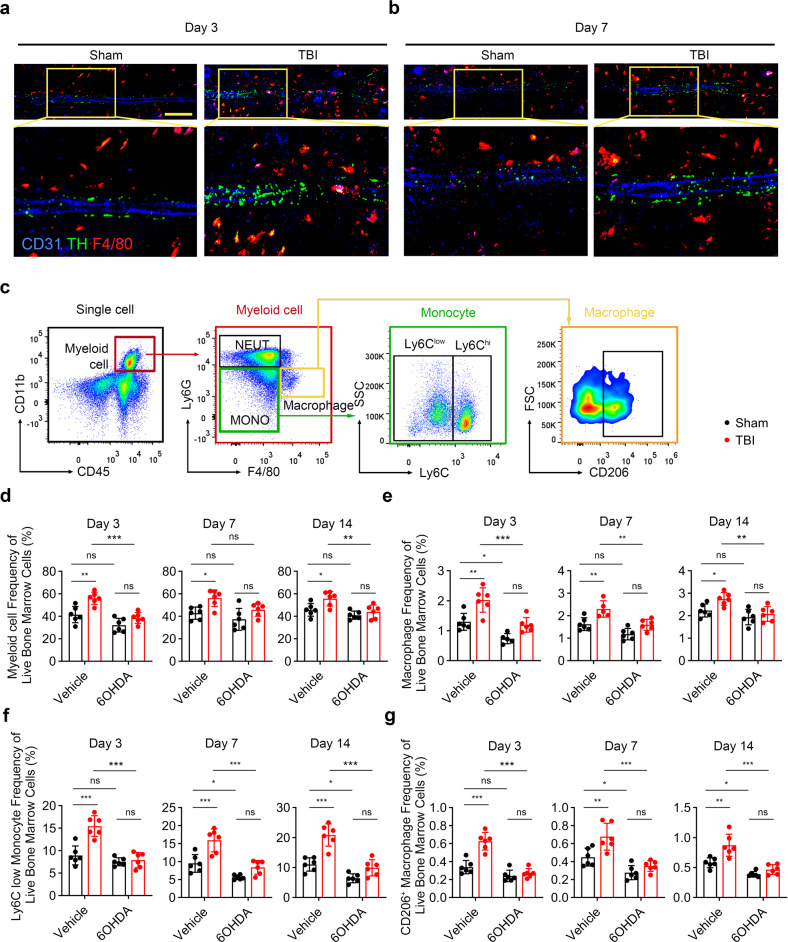
Sympathetic nerve is essential in the expansion of anti-inflammation myeloid cells after TBI. **a**, **b** Representative IF staining images of TH^+^ sympathetic noradrenergic nerve fibers, CD31^+^ endothelial cells, and F4/80^+^ cells in the bone marrow from 4-month-old male fractured mice in sham group, TBI group, and TBI + 6-OHDA group. **c** Flow cytometry plots show gating strategy for myeloid cells (CD45^+^ CD11b^+^), macrophages (CD45^+^ CD11b^+^ Ly6G^−^ F4/80^+^), Ly6C^low^ monocytes (CD45^+^ CD11b^+^ Ly6G^−^ Ly6C^int^), and M2 macrophages (CD45^+^ CD11b^+^ Ly6G^−^ F4/80^+^ CD206^+^). **d**–**g** Quantitative analysis of the myeloid cells, macrophages, Ly6C^low^ monocytes, and M2 macrophages isolated from 4-month-old male mice in sham group, 6-OHDA group, TBI group, and TBI + 6-OHDA group. All data are presented as means ± standard error of the mean (SEM). *P < 0.05, **P < 0.01, and ***P < 0.001, ns: not significant. Statistical significance was determined by two-way ANOVA

Fracture healing is a well-orchestrated immune response process that multiple immune cells play important roles in the inflammatory and repair phase.^45^ To investigate whether TBI induces the expansion of differentiated mature myeloid cells through sympathetic nerves, immunostaining of TH, CD31 and F4/80 were performed to visualize the amount of sympathetic noradrenergic fibers and macrophages in the bone marrow. Significantly increased number of F4/80^+^ cells were found in TBI group compared with that of sham group (Fig. 5a, b). Considering the important role of myeloid cells in fracture healing, we therefore examined the number of anti-inflammation myeloid cells after TBI (Fig. 5c). The number of total myeloid cells and macrophages significantly expanded in TBI group at the 3rd day, 7th day, and 14th day; chemical sympathetic excision diminished the TBI-induced emergency myelopoiesis (Fig. 5d, e and Supplementary Fig. 7a). The monocyte/macrophage lineage derived Ly6G^−^Ter119^−^B220^−^Thy1^−^CD115^+^Ly6C^+^ osteoclast precursors (OCPs) and osteoclasts are important in bone regeneration.^20,46,47^ OCPs contributed to angiogenesis during fracture healing through secreting PDGF-BB.^46,47^ Osteoclasts are essential for the erosion of the cartilage matrix to provide space for osteoblasts to grow into.^20^ The number of OCPs and TRAP^+^ cells significantly increased in callus in the TBI group at the 14th day post fracture. By contrast, treatment of 6-OHDA significantly reduced the counts of OCPs and TRAP^+^ cells in the callus at the 14th day after fracture (Supplementary Figs. 8 and 9). But no significant difference was observed in the number of TRAP^+^ cells and bone mass in the normal femur among the four groups (Supplementary Figs. 9 and 10).

Ly6C^low^ monocytes and alternative activated macrophages are immunoregulative myeloid cells that are important for tissue repair and fracture healing.^48–51^ TBI significantly elevated the frequency of Ly6C^low^ monocytes and CD206^+^ macrophages at the 3rd day, 7th day, and 14th day in bone marrow. Administration of 6-OHDA abrogated the TBI-induced expansion of anti-inflammation myeloid cells (Fig. 5f, g and Supplementary Fig. 7b, c). The number of CD45^+^CD11b^+^F4/80^+^CD206^+^ macrophage in the callus were examined at the 14th day post fracture. The results revealed that the population of M2 macrophages significantly increased in the TBI group. Administration of 6-OHDA diminished the TBI-induced infiltration of M2 macrophages in callus (Supplementary Fig. 11). The serum IL-10 levels significantly increased in the TBI group at 3rd, 7th, and 14th day post fracture. By contrast, the chemical sympathetic excision significantly declined the serum IL-10 levels in TBI-treated mice. TNFα significantly increased in TBI group, but 6-OHDA did not affected the TBI-induced elevating of TNFα. No significant difference in serum levels of IL-4 and IL-1b were observed among these groups at the 7th and 14th day post fracture (Supplementary Fig. 12). Together, these data support that TBI induces expansion of anti-inflammation myeloid cells to regulate the immune environment.

### β2- and β3-adrenergic receptors are essential for TBI-accelerated fracture healing

**Fig. 6:**
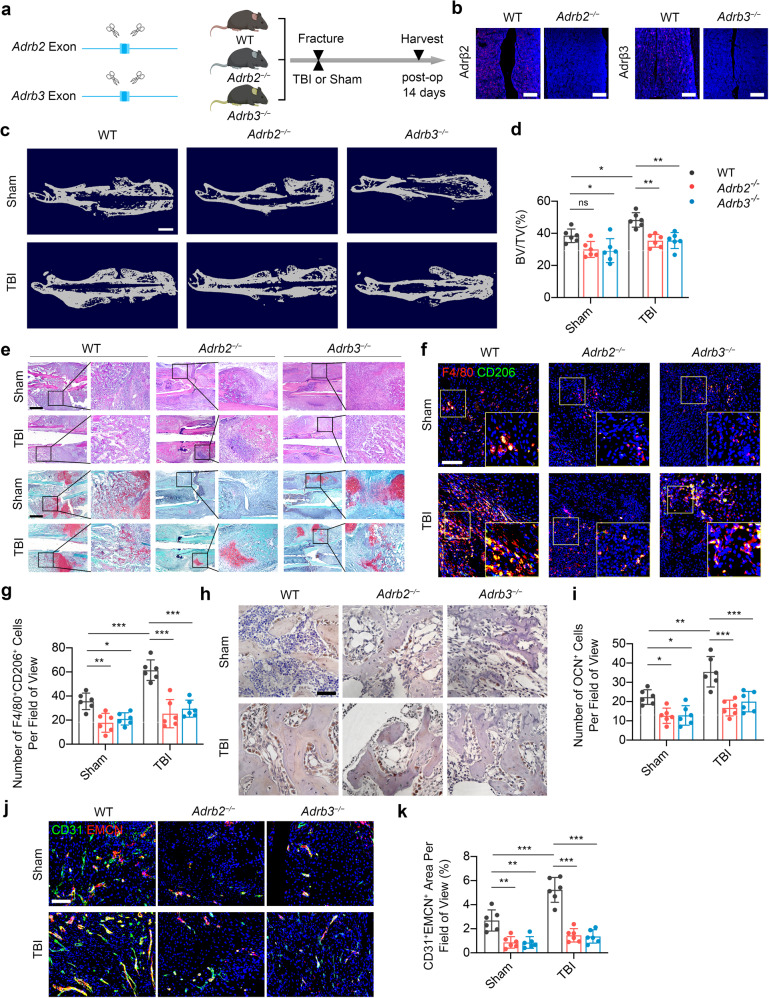
Knockout of *Adrb2* or *Adrb3* compromises the TBI-accelerated fracture healing. **a** Representative IF staining images Ad2β or Ad3β in the bone marrow from 4-month-old *Adrb2*^−/−^ or *Adrb3*^−/−^ mice. **b** Schematic graph of the study of TBI procedure in WT, *Adrb2*^−/−^, and *Adrb3*^−/−^ mice. **c**, **d** Representative *μ*CT images and quantitative analysis of BV/TV of fractured femurs from 4-month-old WT, *Adrb2*^−/−^, and *Adrb3*^−/−^ mice at 14 days post operation. Scale bar: 2 mm. **e** Representative HE and SO/FG staining images in the callus area in 4-month-old male WT, *Adrb2*^−/−^, and *Adrb3*^−/−^ mice. Scale bar: 1 mm. **f**, **g** Representative IF staining images and quantitative analysis of M2 macrophages in the callus area in 4-month-old male WT, *Adrb2*^−/−^, and *Adrb3*^−/−^ mice at 14 days post operation. **h**, **i** Representative immunohistochemical (IHC) staining and quantitative analysis of OCN^+^ cells in the callus from 4-month-old male WT, *Adrb2*^−/−^, and *Adrb3*^−/−^ mice at 14 days post operation. Scale bar: 50 μm. **j**, **k** Representative IF staining images and quantitative analysis of type H vessels in the callus from 4-month-old male WT, *Adrb2*^−/−^, and *Adrb3*^−/−^ mice at 14 days post operation. Scale bar: 200 μm. All data are presented as means ± standard error of the mean (SEM). *P < 0.05, **P < 0.01, and ***P < 0.001, ns: not significant. Statistical significance was determined by two-way ANOVA

β2- and β3-ARs are the most important β-ARs that cooperate hematopoiesis in the bone marrow.^52^ Considering the importance of sympathetic nerves in mediating myelopoiesis as presented above, *Adrb2* deletion (*Adrb2*^*−/−*^) and *Adrb3* deletion (*Adrb3*^*−/−*^) mice were introduced to investigate whether TBI accelerates fracture healing through β2- and β3-ARs (Fig. 6a, b). Compared with the wild type (WT) mice, *Adrb2*^*−/−*^ and *Adrb3*^*−/−*^ mice developed less mineralized callus in TBI group at the 14th day post fracture (Fig. 6c, d). H&E staining and SO/FG staining revealed arrested healing process with persistent fibrous fracture gap in *Adrb2* deletion and *Adrb3* deletion mice in both the sham group and TBI group at the 14th day post fracture (Fig. 6e). IHC analysis revealed that TBI-increased number of the OCN^+^ cells in callus areas were significantly abrogated in mice lacking β2-AR or β3-AR at the 14th day post fracture (Fig. 6f, g). Immunostaining of F4/80 and CD206 revealed that TBI-induced increasing of M2 macrophage infiltrations in callus areas diminished in *Adrb2*^*−/−*^ and *Adrb3*^*−/−*^ mice at the 14th day post fracture (Fig. 6h, i). Accordingly, deletion of *Adrb2* or *Adrb3* abrogated the angiogenesis of type H vessels in callus induced by TBI at the 14th day post fracture, as the evidence showed by immunostaining of CD31 and Endomucin (Fig. 6j, k). These results indicated that both β2 and β3 ARs are essential for TBI-accelerated fracture healing.

### β3-AR signaling promotes HSC proliferation, while β2-AR signaling is required for M2 polarization of macrophages

**Fig. 7:**
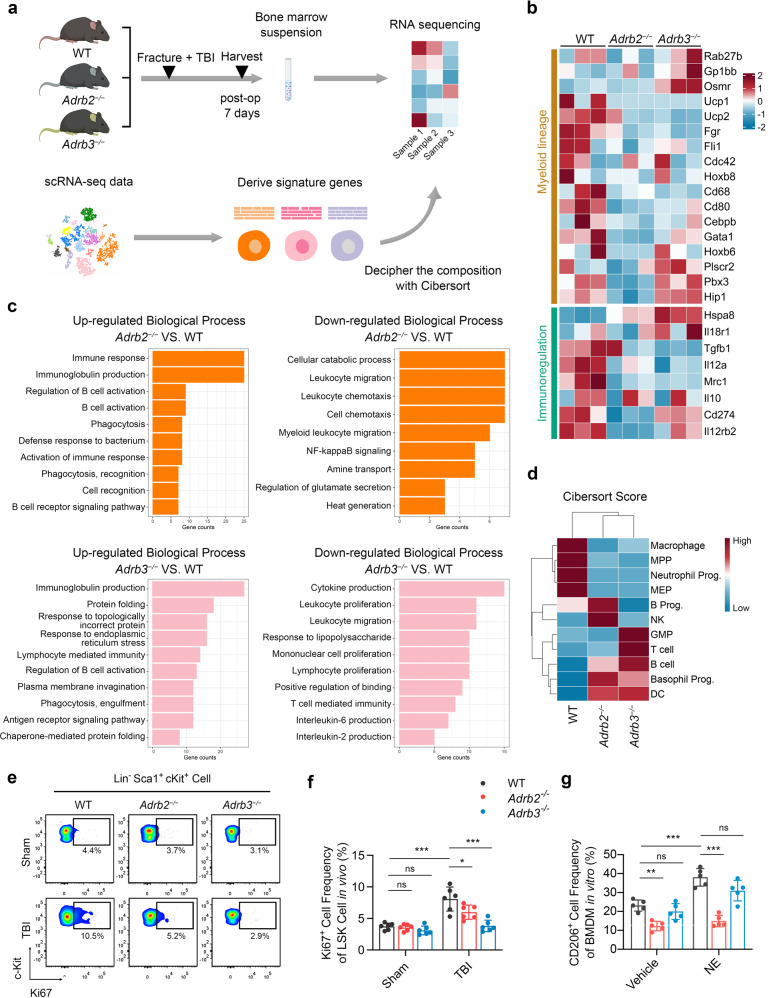
β3 signaling promote HSC proliferation in vivo, while β2 signaling is required for immunoregulation. **a** Schematic graph of the study of decipher the fraction of each cell populations in bone marrow. The bone marrow cells from WT, *Adrb2*^−/−^, and *Adrb3*^−/−^ mice were obtained 7 days after fracture and TBI operation. The signature genes of each cell populations are derived from single-cell RNA sequencing data and are used as reference data to decipher the fraction of cell population in WT, *Adrb2*^−/−^, and *Adrb3*^−/−^ mice. **b** Heatmap of selective gene sets focusing on those reported to be differentially expressed in myeloid cells and immunoregulatory cells. Red represents high expression; blue represents low expression. **c** Bar plot showing the enriched Biological Process enrichment of differentially expressed genes among WT, *Adrb2*^−/−^, and *Adrb3*^−/−^ mice. **d** The fraction scores of each hematopoietic lineage population calculated by Cibersort algorithm. Red represents high score; blue represents low score. **e**, **f** Representative images of flow cytometry and quantitative analysis of proliferative LSKs (Lin^−^ Sca-1^+^ c-Kit^+^ Ki67^+^ cells) isolated from bone marrow of 4-month-old male WT, *Adrb2*^−/−^, and *Adrb3*^−/−^ mice at 7th day post operation. **g** Quantitative analysis of CD206^+^ bone marrow-derived macrophages (BMDM) isolated from WT, *Adrb2*^−/−^, and *Adrb3*^−/−^ mice; the BMDM were treated with vehicle or NE at the presence of IL-4. All data are presented as means ± standard error of the mean (SEM). *P < 0.05, **P < 0.01, and ***P < 0.001, ns: not significant. Statistical significance was determined by two-way ANOVA

To explore the detail mechanisms of β2- and β3-AR signaling in the TBI-induced myelopoiesis, the next-generation sequencing technology was applied to analyze the transcriptomes of bone marrow cells from WT, *Adrb2*^*−/−*^ and *Adrb3*^*−/−*^ mice at the 7th day after TBI combined fracture (Fig. 7a). After obtaining the gene features of each cell populations which were previously described, the frequencies of BM cell types in WT, *Adrb2*^*−/−*^, and *Adrb3*^*−/−*^ mice were evaluated by CIBERSORT algorithm (Fig. 7a).^53^ Selective gene sets that focus on those reported to be differentially expressed in myeloid lineage cells and immunoregulatory cells were evaluated.^14^ Compared with WT mice, decreased levels of myelopoiesis and immunoregulatory of BM cells were observed in both *Adrb2*^*−/−*^ and *Adrb3*^*−/−*^ mice; importantly, BM cells from *Adrb2*^*−/−*^ mice displayed lower expression of myelopoiesis and immunoregulatory related genes compared with the BM cells from *Adrb3*^*−/−*^ mice (Fig. 7b). Top differentially expressed genes between BM cells from *Adrb2*^*−/−*^ mice and WT mice were analyzed through Gene Ontology enrichment of biological process (BP). The upregulated genes in BM cells from *Adrb2*^*−/−*^ mice were found to be related to immune response, B-cell activation, and phagocytosis. Phagocytosis- and lymphocyte-mediated immunity-related genes were upregulated in BM cells from *Adrb3*^*−/−*^ mice compared with WT mice (Fig. 7c). These results suggest the pro-inflammation microenvironment in both *Adrb2*^*−/−*^ and *Adrb3*^*−/−*^ mice after fracture compared with WT mice. Deciphering the frequencies of BM cell populations suggests that the frequency scores of multipotent progenitors (MPPs), megakaryocyte–erythroid progenitors, and macrophages, decreased in BM from *Adrb2*^*−/−*^ mice and *Adrb3*^*−/−*^ mice compared with WT mice. Accordingly, the frequency scores of lymphocytes such as B cells and T cells elevated in *Adrb2*- and *Adrb3*-deficient mice compared with WT mice (Fig. 7d). These results indicate immunoregulation and emergency myelopoiesis were impaired in mice that lack β2-AR or β3-AR in the context of fracture with concomitant TBI.

To further validate the previous findings, we examined the number of BM hematopoiesis lineage cells in the mice that were performed TBI and fracture models (Supplementary Fig. 13a). TBI-induced expansion of LSKs significantly decreased in *Adrb3*^*−/−*^ mice. Slight decline of TBI-expanded LSKs was observed in *Adrb2*^*−/−*^ mice compared with WT mice, but there were no significant differences between the two groups (Supplementary Fig. 13b,c). Accordingly, TBI significantly increases the number of Ki67^+^ LSKs in both WT and *Adrb2*^*−/−*^ mice; while deletion of *Adrb3* significantly abrogated the TBI-induced increase in the number of Ki67^+^ LSKs (Fig. 7e, f). Unexpectedly, there were no significant changes in the number of Ki67^+^ HSCs in the NE-treated group compared with the vehicle group in vitro (Supplementary Fig. 13d,e). Based on these results, we concluded that the TBI induces the proliferation of HSCs mainly through β3-ARs, which requires the microenvironment in bone marrow. We found that TBI increased the M2 polarization of macrophages through sympathetic nerves. To clarify which β-AR mediates the M2 polarization of macrophages, the bone marrow-derived macrophages (BMDMs) from WT, *Adrb2*^*−/−*^, and *Adrb3*^*−/−*^ mice, were treated with IL-4 and were cultured in the presence or absence of NE. NE significantly increased the M2 polarization rates of BMDMs; while deletion of *Adrb2* arrested NE-induced M2 polarization, as evidenced by the decreased proportion of CD206^+^ BMDMs from *Adrb2*^*−/−*^ mice compared with WT and *Adrb3*^*−/−*^ mice (Fig. 7g and Supplementary Fig. 13f). This result reveals that β2-ARs directly mediate the M2 polarization of macrophages. In addition, the TBI-induced expansion of GMPs in bone marrow significantly decreased in both *Adrb2*^*−/−*^ mice and *Adrb3*^*−/−*^ mice at the 7th day post fracture, indicating that both β2-ARs and β3-ARs are essential in the emergency myelopoiesis (Supplementary Fig. 13g). Taken together, our results suggest that the proliferation of hematopoietic progenitors are mainly regulated by β3-ARs, and β2-ARs mediate the M2 polarization of macrophages during fracture healing.

### Selective adrenergic stimulation drugs accelerate fracture healing

**Fig. 8:**
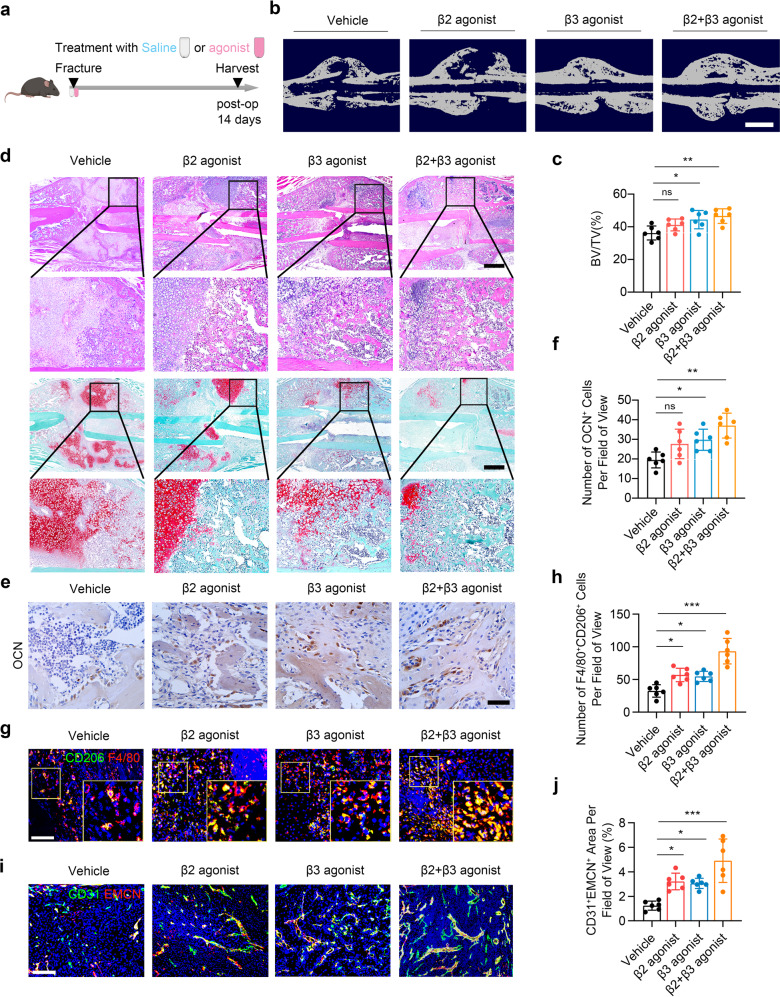
β2-AR and β3-AR agonists synergistically accelerate fracture healing. **a** Schematic graph of the study of β2-AR and β3-AR agonists administration. **b**, **c** Representative *μ*CT images and quantitative analysis of BV/TV of fractured femurs from 4-month-old mice treated with β2-AR agonist, β3-AR agonist, or β2-AR agonist + β3-AR agonist. Scale bar: 2 mm. **d** Representative HE and SO/FG staining images in the callus area from 4-month-old mice treated with β2-AR agonist, β3-AR agonist, or β2-AR agonist + β3-AR agonist. Scale bar: 800 μm. **e**, **f** Representative IF staining images and quantitative analysis of M2 macrophages in the callus area from 4-month-old mice treated with β2-AR agonist, β3-AR agonist, or β2-AR agonist + β3-AR agonist. **g**, **h** Representative immunohistochemical (IHC) staining and quantitative analysis of OCN^+^ cells in the callus from 4-month-old mice treated with β2-AR agonist, β3-AR agonist, or β2-AR agonist + β3-AR agonist. Scale bar: 50 μm. **i**, **j** Representative IF staining images and quantitative analysis of type H vessels in the callus from 4-month-old mice treated with β2-AR agonist, β3-AR agonist, or β2-AR agonist + β3-AR agonist. Scale bar: 200 μm. All data are presented as means ± standard error of the mean (SEM). *P < 0.05, **P < 0.01 and ***P < 0.001, ns: not significant. Statistical significance was determined by one-way ANOVA

Our findings above showed that adrenergic signaling promote fracture healing by stimulating the expansion of macrophages. To explore this finding from a gain-of-function point of view, the selective β2-AR agonist clenbuterol, selective β3-AR agonist BRL37344, and clenbuterol with BRL37344 (cocktail group) was administrated for 14 days, respectively, in the mice performed with fracture (Fig. 8a). Compared to the vehicle group, the BRL37344 group and cocktail group exhibited significantly elevated mineralization rate, as evidenced by the BV/TV through *μ*CT scanning; while there was no significant change in BV/TV between vehicle group and clenbuterol group (Fig. 8b, c). As shown by H&E and SOFG staining, the BRL37344 group and cocktail group displayed abundant area of osteoid and bone in the callus (Fig. 8d). IHC analysis of callus revealed significantly increased expression of OCN in the BRL37344 group and cocktail group (Fig. 8e, f). Immunostaining the callus with F4/80 and CD206 showed a significantly elevated number of M2 macrophages in the clenbuterol, BRL37344, and cocktail group compared with the vehicle group (Fig. 8g, h). Indeed, the area of type H vessels significantly increased in the clenbuterol, BRL37344, and cocktail group (Fig. 8i, j). In summary, these findings reveal that activation of β2- and β3-ARs contributes to the infiltration of M2 macrophages, which orchestrates fracture repairing.

## Discussion

TBI accelerates bone healing has been observed during clinical practice; however, whether there is a direct association between the injured CNS and accelerated bone healing remains unknown. We found that the expression of TH, the rate-limiting enzyme in the synthesis of catecholamines, significantly upregulated in PVN in response to TBI. We identified that the NE levels elevated in TBI mice through a hypothalamus-sympathetic nerve descending neural pathway. These data together suggested the TBI-induced adrenergic hypersensitivity, which mobilized emergency hematopoiesis and led to myeloid bias. We observed increased number of anti-inflammatory myeloid cells, which promote tissue repairing, at the 7th day and 14th day in the bone marrow and callus in TBI group.^48,51^ Moreover, deletion of *Adrb2* or *Adrb3* arrested TBI-accelerated fracture healing. Administration of selective β2-AR agonist clenbuterol with selective β3-AR agonist BRL37344 contributed to fracture healing. Our results suggest a paradigm of hypothalamus-sympathetic nerve descending pathway-induced acute myelopoiesis and anti-inflammation microenvironment in callus during TBI combined fracture.

SNS innervates peripheral organs to prepare the body for ‘fight or flight’ responses and maintains physical homeostasis.^54^ SNS regulates bone remodeling has been well-defined in our previous studies.^29,31,55^ Importantly, sympathetic nerves contribute to the suppression of hyperinflammation and promote tissue repairing process.^36,56^ As TBI increases sympathetic tone after ictus, we examined whether TBI promotes fracture healing through adrenergic hypersensitivity. We reported that chemical excision of sympathetic nerves arrested the TBI-accelerated fracture healing process. Importantly, administration of clenbuterol with BRL37344 synergistically promoted fracture healing. These findings indicate the important role of sympathetic nerves in TBI-accelerated bone regeneration. In line with our study, leukocyte-expressed β2-ARs are essential for tissue recovery after acute myocardial injury.^57^ On the other hand, 8-weeks-chronic stress induces abnormal high sympathetic tone, which leads to bone loss.^58–61^ By contrast, the accelerated fracture healing processes were observed at 7th day and 14th day after TBI, which are relative short terms with intense elevation of sympathetic tone. The contradictory effects of adrenergic signaling have also been reported in the heart. Chronic activation of β2-ARs contributes to the development of heart failure; while β2-ARs-dependent leukocyte recruitment are essential for cardiovascular regeneration after acute injury.^37^ Therefore, the dual role of sympathetic tone in bone formation might be attributed to the duration of adrenergic hypersensitivity. These results suggest that acutely elevated sympathetic tone contributes to the TBI-accelerated fracture healing.

HSCs reside in a specialized microenvironment in the bone marrow, which represents a critical regulatory mechanism by adrenergic signaling to maintaining lifelong blood production.^33^ The self-renew and differentiation of HSCs relies on the innervation of SNS in bone marrow. Specifically, loss the β3-ARs signaling in the bone marrow leads to HSCs aging, which impairs the physiological function of hematopoietic lineage cells.^14^ SNS also mediates the development of inflammatory myeloid cells from CMPs and GMPs.^35^ We found that TBI induces the expansion of the hematopoiesis and myeloid bias through elevated sympathetic tone 3 days, 7 days, and 14 days post fracture. Among mature myeloid cells, Ly6C^low^ patrolling monocytes and F4/80^+^CD206^+^ macrophages are immunoregulatory myeloid cells that are essential for tissue repair.^22,48^ We observed robust Ly6C^low^ patrolling monocytes as well as increased number of F4/80^+^CD206^+^ macrophages at the 7th day and 14th day after fracture in bone marrow in TBI group. TBI elevated the number of M2 macrophages in the callus at the 7th day and 14th day through sympathetic nerves; indeed, selective β2-AR agonist and β3-AR agonist synergistically promoted the infiltration of M2 macrophages in the callus at the 14th day after fracture. Moreover, NE increased the M2 polarization of BMDMs in vitro. These findings are consistent with the NE-induced immunosuppression in clinical practice.^62,63^ However, the regulation of sympathetic nerves to the immune system remains debated. Although short-term administration of NE suppresses the inflammation response,^64^ constant subthreshold stress-elevated sympathetic tone induces egress of Ly6C^hi^ monocytes, which lead to inflammatory environment.^65^ Therefore, the contradictory effects of NE in immune system might owing to the degree and duration of adrenergic hypersensitivity. In summary, these results suggest that TBI shapes the anti-inflammation environment in bone marrow and callus within 14 days via elevated sympathetic tone in mice.

HSC lineage commitments are regulated by β2- and β3-ARs. SNS controls the development of myeloid cells from their progenitors through β2-adrenergic signaling.^34,35^ Inhibition of β3-ARs also alleviated the sympathetic signaling induced myeloid bias in inflammatory conditions.^51,66^ Whereas, NE suppresses the proinflammatory cytokines secretion in human macrophages through β-ARs.^67^ Indeed, the specific roles of β2- and β3-ARs in TBI-accelerated fracture healing have not been explored. Here, we showed that *Adrb2* and *Adrb3* are essential in TBI-accelerated fracture healing. Deletion of *Adrb2* or *Adrb3* diminished the TBI-mediated mineralization, infiltration of macrophages, and angiogenesis of type H vessels at the 7th day and 14th day post fracture. Although a slight increase of mineralization was observed in selective β2-AR agonist clenbuterol group, there was no significant change in BV/TV compared with vehicle group at the 14th day. The sole activation of β2-ARs might not be enough to activate the hematopoietic response to accelerate fracture healing. β3-ARs-mediated emergency hematopoiesis are essential for the expansion of myeloid cells after acute intracerebral hemorrhage.^51^ Therefore, selective β3-AR agonist BRL37344 was introduced. Treatment of BRL37344 with clenbuterol or BRL37344 alone accelerated the fracture healing process at the 14th day, which coupled with the findings that ARs agonists promote tissue regeneration after acute heart injury or intracerebral hemorrhage.^51,57^ RNA-seq data indicated that both β2- and β3-ARs contribute to myelopoiesis and immunoregulation in the context of fracture with concomitant TBI. Importantly, β2-ARs tended to play a more important role in immunoregulation compared with β3-ARs. Flow cytometry further confirmed that deletion of *Adrb2*, not *Adrb3*, arrested NE-induce M2 polarization of BMDMs in vitro. Moreover, deletion of *Adrb3* abrogated the TBI-mediated proliferation of hematopoietic progenitor cells at 7th day post fracture. Therefore, β3-ARs appear to contribute to the expansion of hematopoietic progenitor cells; while β2-ARs shape the anti-inflammation microenvironment through M2 polarization of BMDMs.

**Fig. 9:**
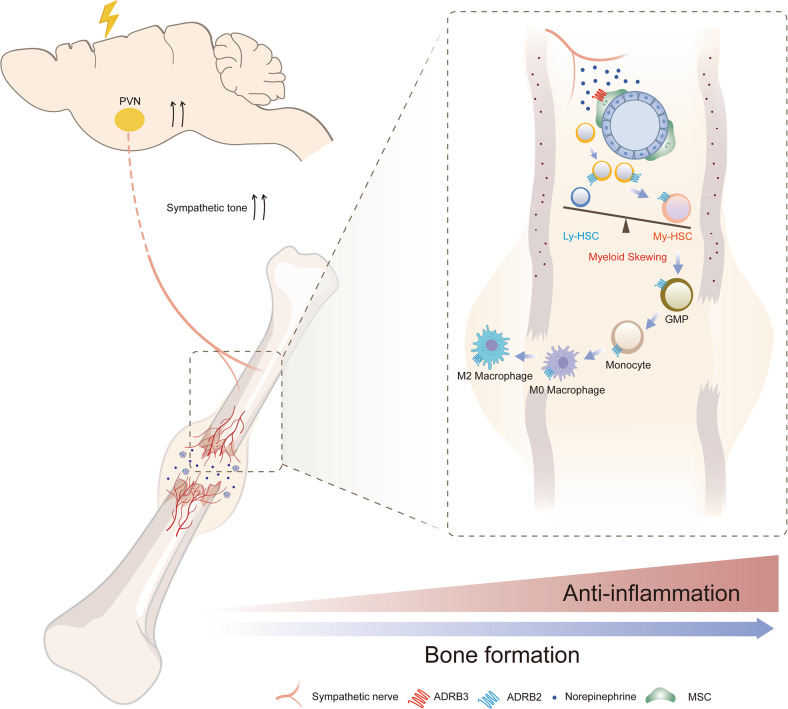
Graphic illustration of this study. Traumatic brain injury (TBI) activates hypothalamic paraventricular nucleus (PVN) to increase the sympathetic outflow. The elevated sympathetic tones trigger the proliferation of hematopoietic stem cells through β3-adrenergic receptor (ARs) in bone marrow. Importantly, the sympathetic hypersensitivity induced activation of β2-adrenergic signaling, which promotes myelopoiesis and M2 polarization of macrophages in callus. During the early stage of fracture healing, TBI-induced sympathetic hypersensitivity facilitates the anti-inflammation microenvironment in callus by activating β adrenergic receptors, therefore accelerates the fracture healing process. MSC mesenchymal stem cell, Ly-HSC lymphoid-biased hematopoietic stem cell, My-HSC myeloid-biased hematopoietic stem cell, GMP granulocyte monocyte progenitor

This study has identified serval therapeutic targets in fracture healing, such as bone marrow sympathetic nerves, β2- and β3-ARs. We recognize the possibility of off-target effects. Therefore, optimized strategy that harnessing the hematopoietic response to sympathetic signaling for trauma recovery remains to be developed. In addition, sympathetic nerves were found to directly regulates the activities of bone marrow MSC in our previous studies.^29,55^ As periosteal stem cell is crucial for fracture healing,^68^ future studies will be required to investigate whether adrenergic signaling regulates periosteal stem cell during fracture. In conclusion, we offer a view of neuron-hematopoiesis interactions during fracture healing in the context of concomitant TBI. Our results suggest that the injured CNS elevates sympathetic tone to mobilize HSCs lineage cells and to promotes myeloid bias. These expanded myeloid cells suppress the inflammation in callus to accelerate fracture healing (Fig. 9). Recognition of this mechanism will contribute to developing fracture healing therapies by harnessing beneficial hematopoiesis.

## Materials and methods

### Ethics approval

In this study, all the experiments were approved by the local ethical committees. The inclusion of human subjects and supporting documentation was approved by the Ethics Committees of Union Hospital, Tongji Medical College, Huazhong University of Science and Technology (2022-0245). All patients signed the informed consents before they were enrolled in the study. All animal experiments were approved by Experimental Animal Ethics Committee of Hebei Ex & In vivo Biotechnology Co., Ltd. (SY2020-01). The experiments were performed in accordance with the Animal Research: Reporting In Vivo Experiments guidelines.

### Study design

The major goal of this study was to explore the underlying mechanism of accelerated fracture healing in patients combined with TBI. To verify whether patients with TBI exhibited accelerated fracture healing than the isolated fracture, the following-up data of 41 fracture patients (20 patients with isolated fracture and 21 patients suffered from fracture and TBI) were collected and analyzed. Serum was obtained from 22 male patients (14 with femur fractures, 8 with femur fracture combined with TBI) without underlying disease via venous blood draw when the patients were admitted to the emergency room in the Union Hospital, Tongji Medical College, Huazhong University of Science and Technology. To investigate whether TBI promote fracture healing through toning the sympathetic activity, 6-OHDA was introduced for chemical sympathetic excision. Mice subjected to fracture were divided into four groups: sham operation group, TBI group, sham operation + 6-OHDA group, TBI + 6-OHDA group. The callus tissues were collected to detect the fracture healing rate. Bone marrow cells were collected to analyze the differentiation of HSCs. To characterize the role of sympathetic signal in fracture healing and hematopoietic mobilization, the mice were subjected to fracture and were treated with vehicle, norepinephrine, clenbuterol (β2-adrenergic agonists) or BRL37344 (β3-adrenergic agonists). The detailed values of sample size are provided in results part or figure legends. All experiments have been replicated for at least three times.

### Mice

All studies were performed in adult male mice of 4 months old. C57BL/6J were purchased from Animal center of Hebei Ex & In vivo Biotechnology Co., Ltd. *Adrb2*^*−/−*^, *Adrb3*^*−/−*^ mice were purchased from Shanghai Model Organisms Center, Inc., and were backcrossed to C57BL/6J background in our laboratory. The genotypes of the mice were determined by PCR of genomic DNA that extracted from mouse tails with the following primers:

*Adrb2*

forward: P1 TGTGGCTGTGGTTCGGCATAAGTC,

reverse: P1 AAGGGCCCATTGTCACAGCAGAAA;

forward: P2 TTGGCCCAAAGTTGTTGCAC,

reverse: P2 GCAAGAAGTCGCTGTCGTTC;

*Adrb3*

forward; P1 CAGGTTCTGCCAGGAAGGAG,

reverse: P1 AGCACTGGAAGGAAGAGGGA;

forward: P2 CTCCACCCTCCAATTCCCAC,

reverse: P2 ATTACCAGCAGGTTGCCTCC.

All mice were maintained under pathogen-free conditions with standardized light-dark cycle conditions. All mice were housed with other three to four mice per cage.

### Animal models

TBI was performed using a weight-drop device as described previously.^9^ Briefly, the mice were anesthetized via intraperitoneal injection of ketamine/xylazine and were fixed on a stereotaxic apparatus. After exposure of the cranium, a 4 mm diameter hole was drilled at the left side of the skull to allow placing a T-shaped firing pin with a depth of 3 mm. Then a 20 g weight was dropped onto this area from 20 cm above. The incision was then sutured with silk sutures. The mouse neurological severity score (mNSS) system was introduced to assess the degree of brain injury. The brain tissues were harvested at 7 days or 14 days post-surgery for sectioning and staining.

The bone fracture procedure was performed as described in our previous article.^31^ Briefly, a 25-gauge stainless steel pin was inserted in right femur from the distal femur into the intramedullary canal. Fracture was made by an electric diamond disk; the surgery region was then sutured layer by layer. Thereafter, the mice were transferred into cages after they recovered from surgery. All mice were checked every day after surgery. The callus and contralateral normal femurs were harvested at 7 days or 14 days post-surgery. Micro-CT, bone sectioning, H&E staining, immunohistochemistry staining, and immunofluorescence staining were further performed on these bone samples.

### In vitro culture assays

For bone marrow-derived macrophages (BMDMs) culturing, femurs were flushed to get bone marrow cells. The bone marrow cells were cultured with DMEM medium that containing 10% FBS, 1% penicillin–streptomycin (Solarbio, P1400) and 20 ng/ml m-CSF (R&D, 416-M-050). The medium was refreshed at day 5 and added with 20 ng/ml of IL-4 for 4 days to induce M2-like BMDMs, during which vehicle or 10 μM NE was added to the culture. For in vitro analyses of HSCs, bone marrow cells were treated with ACK lysing buffer (BD Biosciences) for 2 min to exclude red blood cells. Then the lineage-depleted bone marrow cells were obtained through Hematopoietic Cell Lineage Depletion Kit (R&D Systems, MAGM209). The lineage-depleted bone marrow cells were cultured for 7 days in StemSpan medium (StemCell Technologies) containing SCF (R&D Systems, 445-MC, 10 ng/ml), Fgf1 (R&D Systems, 4686-FA, 10 ng/ml) and Thpo (R&D Systems, 488-TO, 20 ng/ml).^69^ Vehicle or 10 μM NE was added to assess NE-related effects on HSCs.

### In vivo drug administration

For chemical sympathetic neuronal ablation, mice were treated with 6-OHDA (100 mg/Kg at day 1 and 250 mg/Kg at day 3) by intraperitoneal injection two days before TBI. Another injection of 6-OHDA at the dose of 250 mg/Kg was performed at 8th day after TBI. Long-term administration of norepinephrine (Sigma, 5 ug/Kg/min), clenbuterol (Sigma, 2 mg/kg/day) and BRL37344 (Sigma, 2.4 mg/kg/day) were performed by Alzet osmotic pumps (model 2001 for 3–7 days and model 2002 for 14 days; DURECT Corporation). Control animals were implanted with Alzet pumps containing saline.

### Histology, immunohistochemistry, and immunofluorescence assay

Brain and bone tissues were collected and fixed in 4% paraformaldehyde overnight. Bone tissues were decalcified by using 0.5 M EDTA (pH 7.26) at 4 °C for 14 days. These fixed samples were dehydrated with 30% sucrose and were embedded in paraffin or optimal cutting temperature compound (Sakura Finetek). Four-micrometer-thick sections were prepared for H&E (Solarbio, G1121), SO/FG (Servicebio, G1053), TRAP (Servicebio, G1050) and immunohistochemistry staining. Histology staining were performed according to the manufacturer’s protocols. Ten-micrometer-thick sections were prepared for brain tissues. Twenty-micrometer-thick coronal sections of the bone tissues were obtained for macrophage and vessel staining, and 40-μm-thick sections for sympathetic nerve staining. Immunostaining was performed using standard protocols. Briefly, for sympathetic nerve staining, the sections were incubated with primary antibodies against TH (MilliporeSigma, AB152; 1:400) at 4 °C for 72 h. For other antigens, the sections were incubated with primary antibodies overnight at 4 °C. The antibodies we used were as follows: OCN (Takara Bio, M188, 1:200), Osterix (Abcam, ab22552; 1:100), F4/80 (Invitrogen, MA1-91124; 1:100), CD206 (Abcam, ab64693; 1:1000), CD31 (R&D Systems, AF3628; 1:100) and Endomucin (Santa Cruz, sc-65495; 1:50). An HRP-streptavidin detection kit (ZSGB-Bio; SP-9000) was used in immunohistochemical procedures, followed by counterstaining with Mayer’s hematoxylin solution (Solarbio, G1080). Fluorescence-conjugated secondary antibodies were used as follows in immunofluorescence procedures: donkey anti-goat IgG H&L (Alexa Fluor 405) (Abcam, ab175664; 1:100), donkey anti-goat IgG (H + L) FITC (Invitrogen, A16000; 1:500), donkey anti-rabbit IgG (H + L) (Alexa Fluor 488) (Invitrogen, A-21206; 1:500), donkey anti-rabbit IgG (H + L) TRITC (Invitrogen, A16026; 1:500), donkey anti-rat IgG (H + L) TRITC (Invitrogen, A18744; 1:500). Olympus FV1200MPE confocal microscope or Olympus BX51 microscope were used for sample image capturing. Quantitative histomorphometric analysis was performed by ImageJ in a blinded fashion.

### Flow cytometry assay

For flow cytometry assay of bone marrow cells, the tibias were dissected, and the bone marrow was obtained by pulsed flushing with 1 mL cold PBS containing 2% FBS. For flow cytometry assay of the callus, the callus was dissected and crushed. Then the bone pieces were further digested by digesting buffer mixture containing α-MEM containing 3 mg/mL collagenase I (Worthington), 4 mg/mL dispase (MilliporeSigma), and 1 U/mL DNAse I (Invitrogen) for 30 min in a shaking water bath at 37 °C. Then the suspension was passed through a 70-μm cell strainer to remove tissue fragments. Thereafter, the suspension was centrifuged at 1500 rpm for 5 min at 4 °C. The sediment was resuspended in ACK lysing buffer (BD Biosciences) for 2 min to exclude red blood cells and was stopped with double volume buffer. The suspension was centrifuged at 1500 rpm for 5 min at 4 °C for another time and the sediment was resuspended in 100 μL cold PBS containing 2% FBS and stained with antibodies for 30 min at 4 °C in the dark. Before intracellular staining, cells were fixed and permeabilized with Fixation and Permeabilization Kit (BD Biosciences, 554714) according to the instruction manual. The antibodies used were as follows: anti-mouse CD45–APC–Cy7 (BD Biosciences, 557659; 1:100), anti-mouse CD11b–FITC (BD Biosciences, 557396; 1:100), anti-mouse Ly6C–PerCP–Cy5.5 (BD Biosciences, 560525; 1:200), anti-mouse Ly6G–eFluor 450 (eBioscience, 48-9668-82; 1:100), anti-mouse F4/80–Super Bright 645 (eBioscience, 64-4801-82; 1:200), anti-mouse CD206–APC (eBioscience, 17-2061-82; 1:150), anti-mouse Lineage–PerCP–Cy5.5 (BD Biosciences, 561317; 1:100), and anti-mouse Sca-1–APC (eBioscience, 17-5981-83; 1:100), anti-mouse CD127 (IL7Rα)–Brilliant Violet 711 (BD Biosciences, 565490; 1:100), anti-mouse CD34–PE (BD Biosciences, 551387; 1:100), anti-mouse CD16/CD32–APC–Cy7 (BD Biosciences, 560541; 1:100), and anti-mouse CD117 (c-Kit)–Brilliant Violet 421 (BD Biosciences, 562609; 1:100), anti-mouse Ki67–Brilliant Violet 786 (BD Biosciences, 563756; 1:50), CD31–APC–Cy7 (Biolegend, 102440; 1:100), CD24–BV605 (Biolegend, 101827; 1:100), B220–BV421 (BD Biosciences, 562922; 1:100), Thy-1.2–BV421 (Thermo, 48-0902-82; 1:100), Ter119–BV421 (Thermo, 48-5921-82; 1:100), CD115–FITC (Thermo, MA5-17859; 1:100). Cells were resuspended in staining buffer before flow cytometry, and were examined on a Sony ID7000 flow cytometer. The results were analyzed using FlowJo version 10.0 (Treestar).

### RNA extraction and library construction

After obtain the bone marrow cells, total RNA was isolated using TRIzol reagent (Invitrogen, Carlsbad, CA, USA) following the manufacturer’s procedure. The quality and amount of RNA from each sample were quantified using NanoDrop ND-1000 (NanoDrop, Wilmington, DE, USA). Dynabeads Oligo (dT) (Thermo Fisher, 25-61005, CA, USA) was used to purify Poly (A) RNA for two rounds of purification. Then the poly(A) RNA was fragmented under 94 °C 5 min. Then the RNA fragments were reverse-transcribed to cDNA by SuperScript™ II Reverse Transcriptase (Invitrogen, cat. 1896649, USA). The library construction was performed with assistance from LC-Bio Technology CO., Ltd. The sequencing was performed on an illumina Novaseq™ 6000 (LC-Bio Technology CO., Ltd., Hangzhou, China) following the vendor’s recommended protocol.

### Real-time fluorescence quantitative PCR

We performed real-time quantitative PCR (qPCR) using the SYBR Green Power PCR Master Mix (Invitrogen, A25777) on a CFX Connect instrument (Bio-Rad); Gapdh was used as an internal control to calculate the relative expression level of mRNA. Sequences of the primers used for each gene were as follows:

*Ucp1*:

forward: CTTTGCCTCACTCAGGATTGG,

reverse: ACTGCCACACCTCCAGTCATT;

*Gapdh*:

forward: ATGTGTCCGTCGTGGATCTGA,

reverse: ATGCCTGCTTCACCACCTTCTT;

*Tnfsf11 (Rankl)*:

forward: CAGAAGGAACTGCAACACAT,

reverse: CAGAGTGACTTTATGGGAACC;

*Tnfrsf11b(Opg)*:

forward: TCCCTTGCCCTGACCACTCTTATAC,

reverse: CCTCACACTCACACACTCGGTTG.

### Bioinformatics analysis of RNA-seq

The fastp software (https://github.com/OpenGene/fastp) was applied for quality control. HISAT2 (https://ccb.jhu.edu/software/hisat2) was used to map reads to the reference genome of Mus musculus GRCm38. The mapped reads of each sample were assembled using StringTie (https://ccb.jhu.edu/software/stringtie) to reconstruct comprehensive transcriptome. The expression level for mRNAs were estimated by calculating fragments per kilobase of exon model per million mapped fragments (FPKM). The differentially expressed genes were selected by R package Limma.

The processed counts matrix of single-cell RNA sequencing data was directly obtained from GSE122467. Data processing of scRNA-seq data was performed by Seurat (version 3.0.1).^70^ After obtaining the signature genes of each cell populations in bone marrow, we applied CibersortX to decipher the composition of each cell subtype in bulk RNA sequencing data of bone marrow cells from WT, *Adrb2*^*−/−*^, and *Adrb3*^*−/−*^ mice. DEGs between WT, *Adrb2*^*−/−*^, and *Adrb3*^*−/−*^ mice were identified by the Limma R package with the cut off threshold at adj. *P* value < 0.05 and fold change >1.5. The DEGs were subsequently used for GO enrichment analysis as well as KEGG analysis with clusterProfiler.

### Enzyme-linked immunosorbent assay

The level of noradrenaline (Arigo, ARG80475), IL-4 (Invitrogen), TNF alpha (Invitrogen), IL-1β (Invitrogen), and IL-10 (Invitrogen) was detected by enzyme-linked immunosorbent assay (ELISA) Kit following the manufacturer’s protocol. The whole-blood samples were obtained after euthanasia by cardiac puncture immediately. Serum was collected by centrifuge at 1500 rpm for 15 min and stored at −80 °C. Extracellular fluids of the tibia were collected from the supernatants following bone marrow extraction in the flow cytometry procedures and stored at −80 °C.

### μCT analyses

Calluses were harvested and the soft tissue around the bone was removed. μCT analyses were performed with SkyScan 1174 μCT scanner. The scanning procedure was performed at 65 kV with a 150-μA current. The resolution was set to 9 μm/pixel. NRecon (version 1.6, SkyScan) was used to reconstruct image, CTAn (version 1.9, SkyScan) was used for quantitative analysis to obtain trabecular bone volume/total volume (Tb. BV/TV), trabecular thickness (Tb. Th), trabecular number (Tb. N), and trabecular separation (Tb. Sp). CTVol (version 2.0, SkyScan) were used to build coronal plane image of the callus.

### Statistics

All data analyses were performed using Prism software, version 8.0 (GraphPad Software, La Jolla, CA). Data are presented in result and figures as mean ± SEM. Two-tailed Student’s *t* tests were used for comparisons between two groups, Chi-square test was used for comparison of two rates or two constituent ratios, and two-way ANOVA was used for comparisons among multiple groups. Results with *P* value less than 0.05 was deemed significant. All representative experiments were repeated at least three times. The data can be provided from the corresponding authors upon reasonable request.

## Supplementary information

Supplementary_Materials

## Data Availability

All data supporting the findings of this study are available within the Article or be publicly available through contacting the corresponding authors. The gene expression omnibus (GEO) accession number for the sequencing data reported in this paper is GSE229258.
