# Supplementary material for: Traumatic brain injury stimulates sympathetic tone-mediated bone marrow myelopoiesis to favor fracture healing
Source: Signal Transduct Target Ther. 2023 Jul 5;8:260. doi: 10.1038/s41392-023-01457-w (PMC10319807; doi:10.1038/s41392-023-01457-w)
Supplement: Supplementary file 1 — Supplementary_Materials [file 41392_2023_1457_MOESM1_ESM.docx]

Supplementary Materials for

Traumatic Brain Injury Stimulates Sympathetic Tone-mediated Bone Marrow Myelopoiesis to Favor Fracture Healing

Weijian Liu, Wei Chen, Mao Xie, Chao Chen, Zengwu Shao, Yiran Zhang, Haiyue Zhao, Qingcheng Song, Hongzhi Hu, Xin Xing, Xianyi Cai, Xiangtian Deng, Xinyan Li, Peng Wang, Guohui Liu, Liming Xiong*, Xiao Lv*, Yingze Zhang*

Correspondence to: yzling_liu@163.com, lvxiaotjmu@163.com and xiongliming@hust.edu.cn

**This PDF file includes:**

Figures. S1 to S13

Tables S1 to S2

Figure. S1.


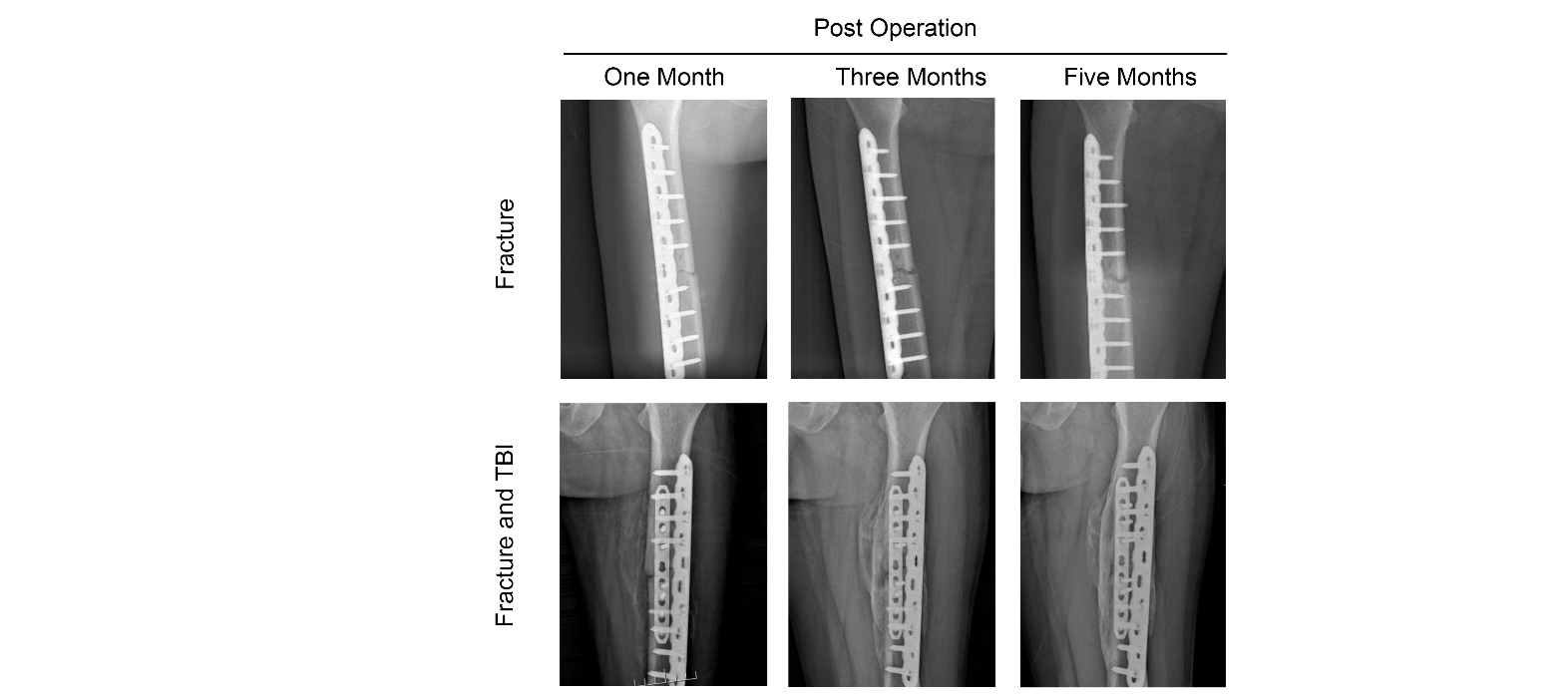


Supplementary Fig. 1 Representative X-ray images in patients with femur fracture or femur fracture subjected with TBI.

Figure. S2.


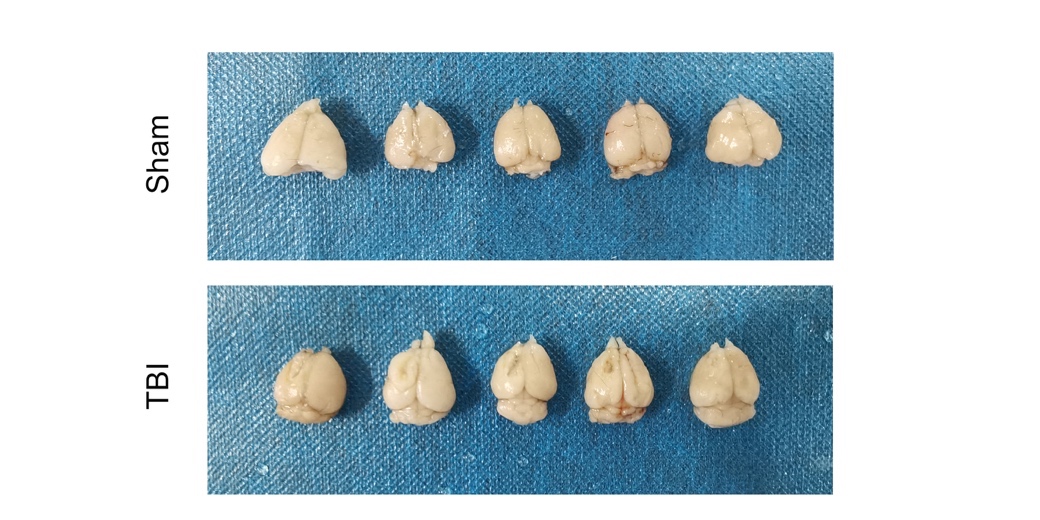


Supplementary Fig. 2 Representative general view of brain tissue from mice in sham group and TBI group.

Figure. S3.


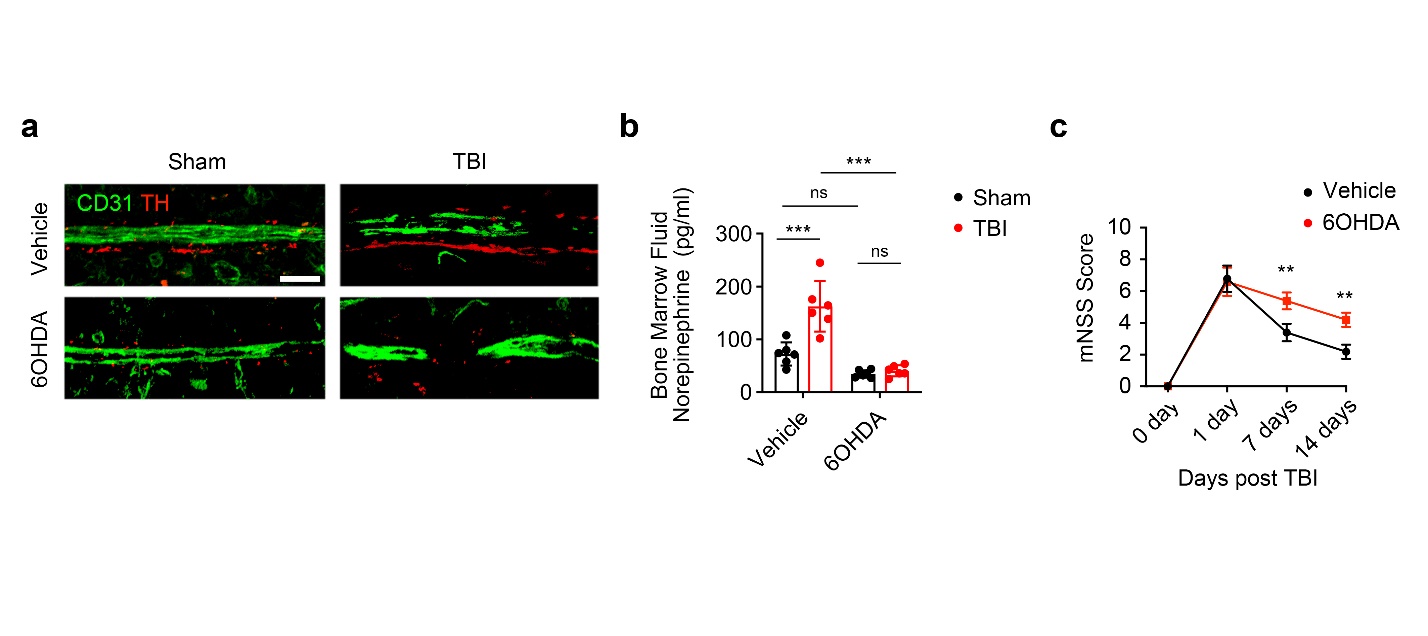


Supplementary Fig. 3 **a** Representative IF staining of TH expression in the bone marrow from 4-month-old male mice in sham group, 6OHDA group, TBI group, and TBI + 6OHDA group at 7^th^ day post operation. Scale bar: 50 μm. **b** Quantitative analysis of bone marrow fluid NE by ELISA assay from 4-month-old male mice in sham group, 6OHDA group, TBI group, and TBI + 6OHDA group at 7^th^ day post operation. **c** Quantitative analysis of mouse NSS score from 4-month-old male mice in TBI group and TBI + 6OHDA group at 1^st^, 7^th^ and 14^th^ day post operation. All data are presented as means ± standard error of the mean (SEM). **p* < 0.05, ***p* < 0.01 and ****p* < 0.001, ns: not significant. Statistical significance was determined by two-tailed Student’s t-test or two-way ANOVA.

Figure. S4.


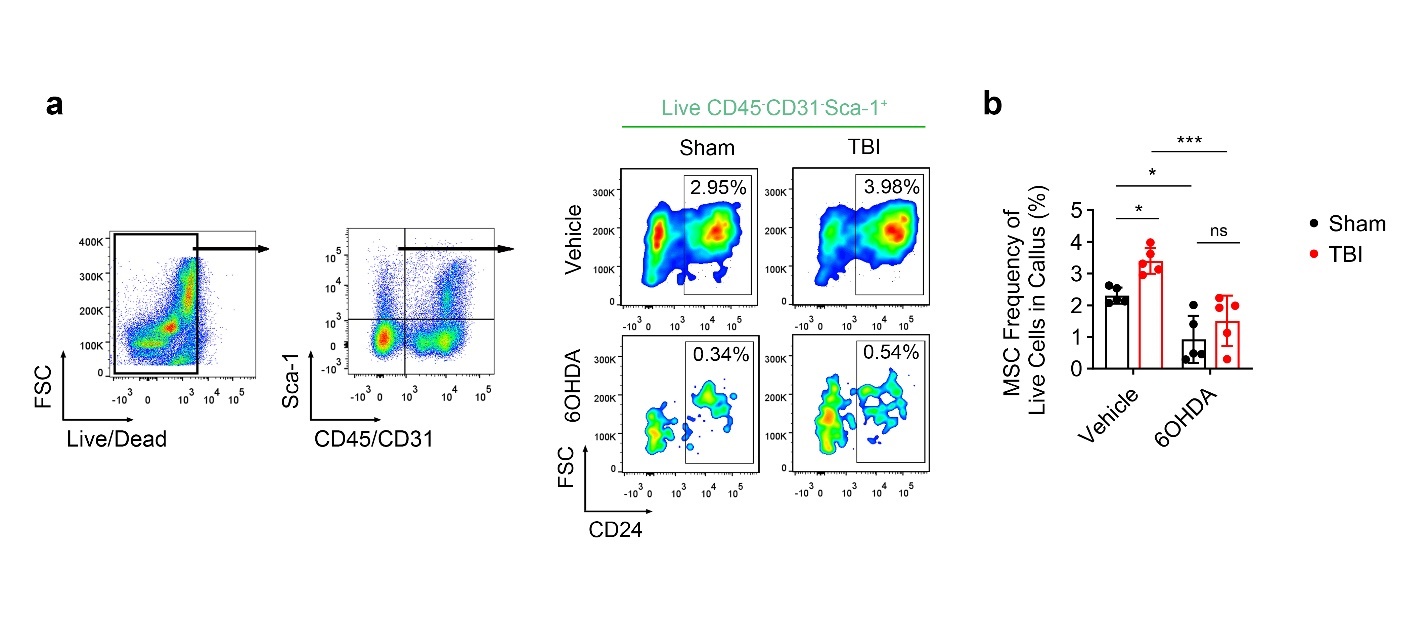


Supplementary Fig. 4 **a**, **b** Representative images of flow cytometry and quantitative analysis of MSCs (Live CD45^–^CD31^–^Sca-1^+^CD24^+^ cells) isolated from callus of 4-month-old male mice in sham group, 6OHDA group, TBI group, and TBI + 6OHDA group at 14^th^ day post operation. All data are presented as means ± standard error of the mean (SEM). **p* < 0.05, ***p* < 0.01 and ****p* < 0.001, ns: not significant. Statistical significance was determined by two-way ANOVA.

Figure. S5.


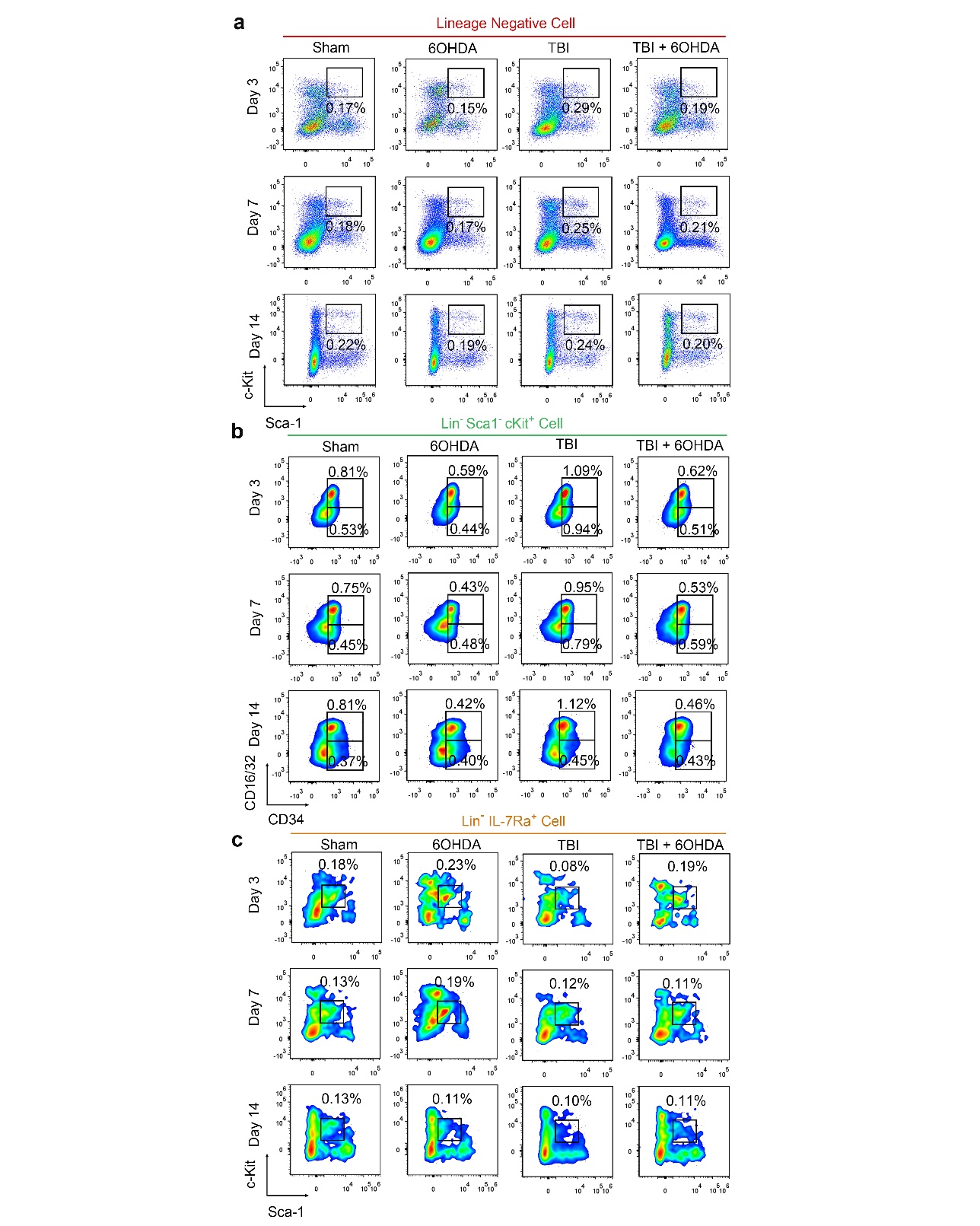


Supplementary Fig. 5 **a-c** Representative flow cytometry plots of Lin^−^ Sca-1^+^ c-Kit^+^ cells, Lin^−^ Sca-1^−^ c-Kit^+^ CD34^+^ CD16/32^int^ cells, Lin^−^ Sca-1^−^ c-Kit^+^ CD34^+^ CD16/32^+^ cells and Lin^−^ Sca-1^int^ c-Kit^int^ IL7R^+^ cells isolated from 4-month-old male mice in sham group, 6OHDA group, TBI group, and TBI + 6OHDA group.

Figure. S6.


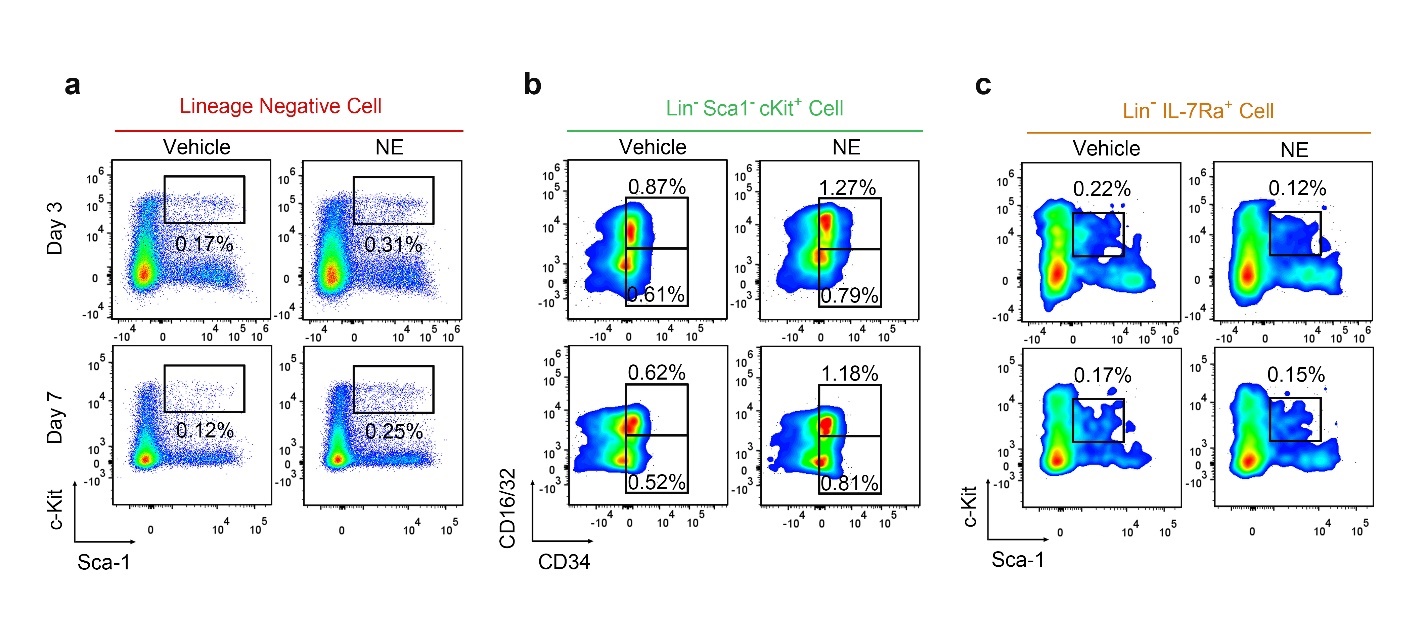


Supplementary Fig. 6 NE promotes bone marrow myelopoiesis after TBI. **a** Representative flow cytometry plots of Lin^−^ Sca-1^+^ c-Kit^+^ cells isolated from 4-month-old male mice treated with vehicle or NE. **b** Representative flow cytometry plots of Lin^−^ Sca-1^−^ c-Kit^+^ CD34^+^ CD16/32^int^ cells and Lin^−^ Sca-1^−^ c-Kit^+^ CD34^+^ CD16/32^+^ cells isolated from 4-month-old male mice treated with vehicle or NE. **c** Representative flow cytometry plots of Lin^−^ Sca-1^int^ c-Kit^int^ IL7R^+^ cells isolated from 4-month-old male mice treated with vehicle or NE.

Figure. S7.

**
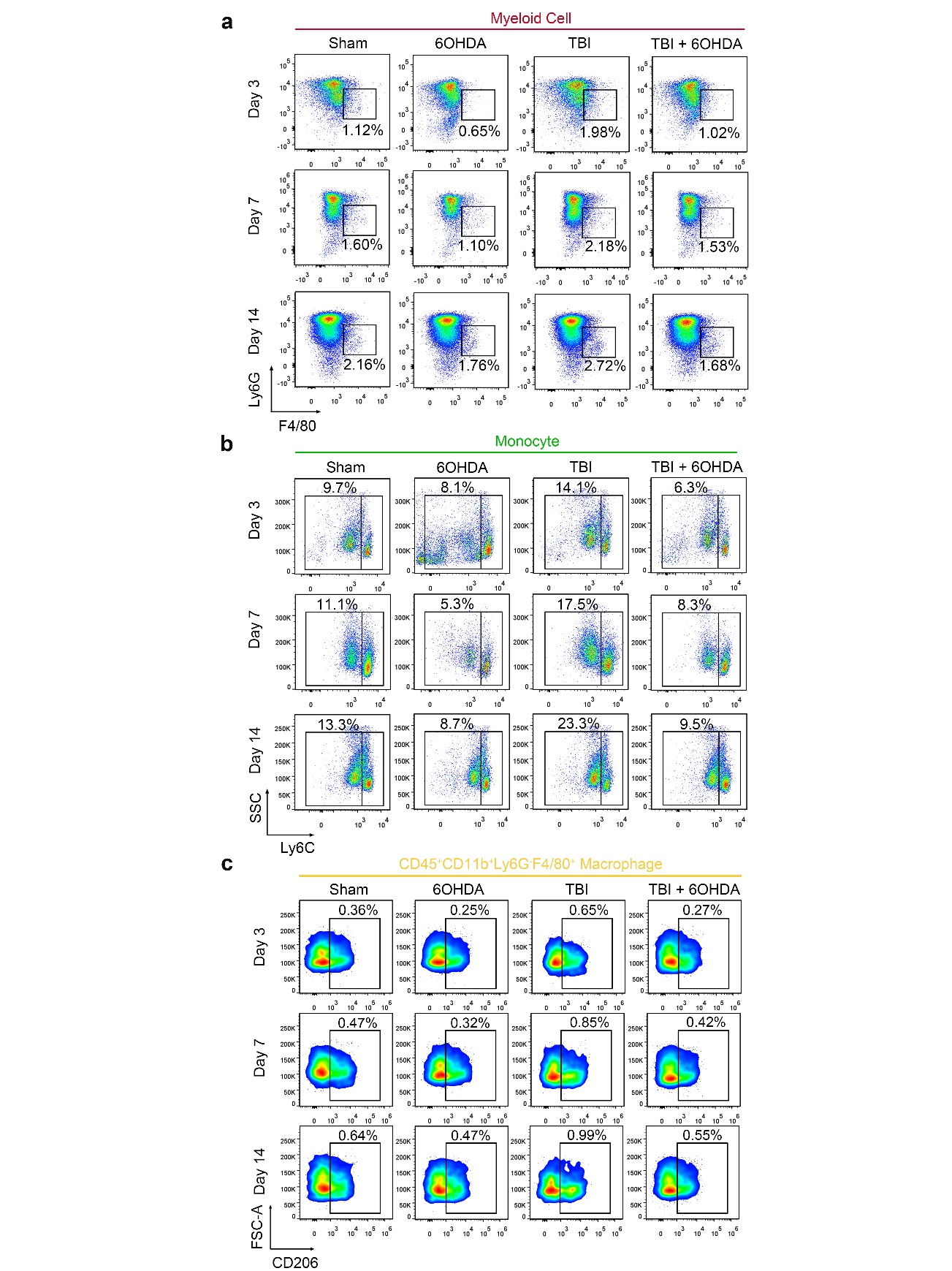
**

Supplementary Fig. 7 **a-c** Representative flow cytometry plots of CD45^+^ CD11b^+^ Ly6G^−^ F4/80^+^ cells, CD45^+^ CD11b^+^ Ly6G^−^ Ly6C^int^ cells and CD45^+^ CD11b^+^ Ly6G^−^ F4/80^+^ CD206^+^ cells isolated from 4-month-old male mice in sham group, 6OHDA group, TBI group, and TBI + 6OHDA group.

Figure. S8.


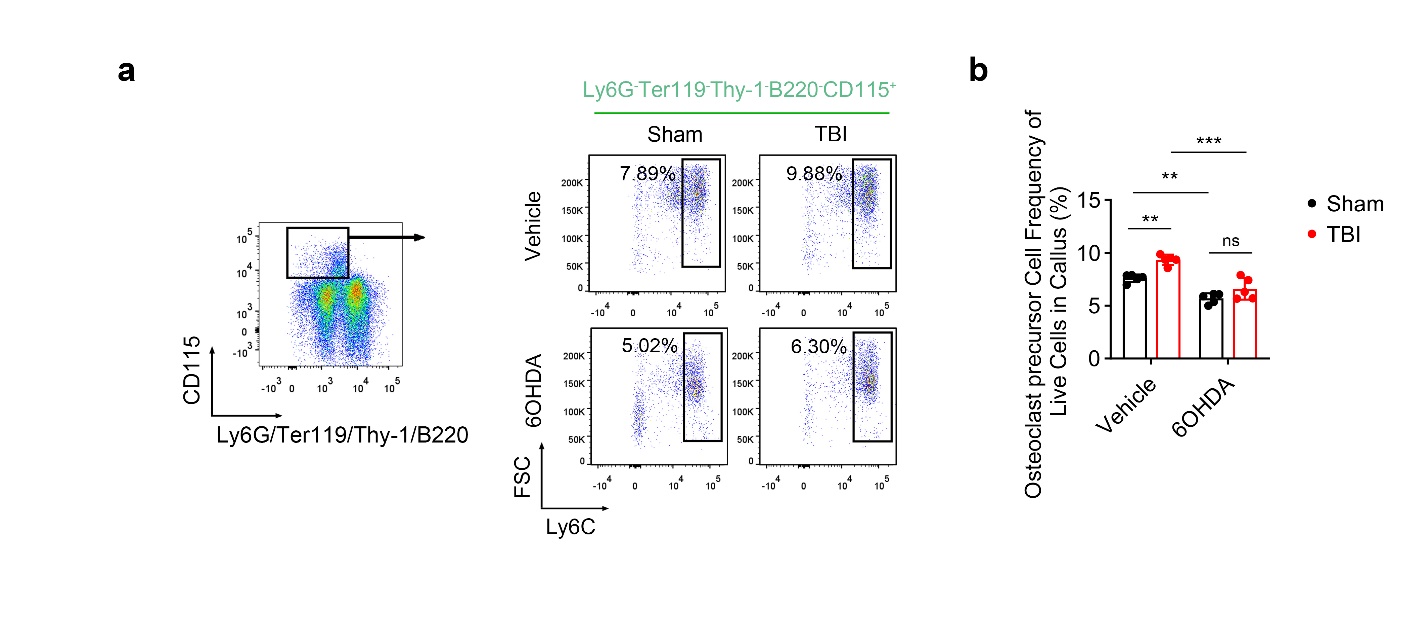


Supplementary Fig. 8 **a, b** Representative images of flow cytometry and quantitative analysis of Ly6G^−^Ter119^−^B220^−^Thy1^−^CD115^+^Ly6C^+^ osteoclast precursors (OCPs) isolated from callus of 4-month-old male mice in sham group, 6OHDA group, TBI group, and TBI + 6OHDA group at 14^th^ day post operation. All data are presented as means ± standard error of the mean (SEM). **p* < 0.05, ***p* < 0.01 and ****p* < 0.001, ns: not significant. Statistical significance was determined by two-way ANOVA.

Figure. S9.


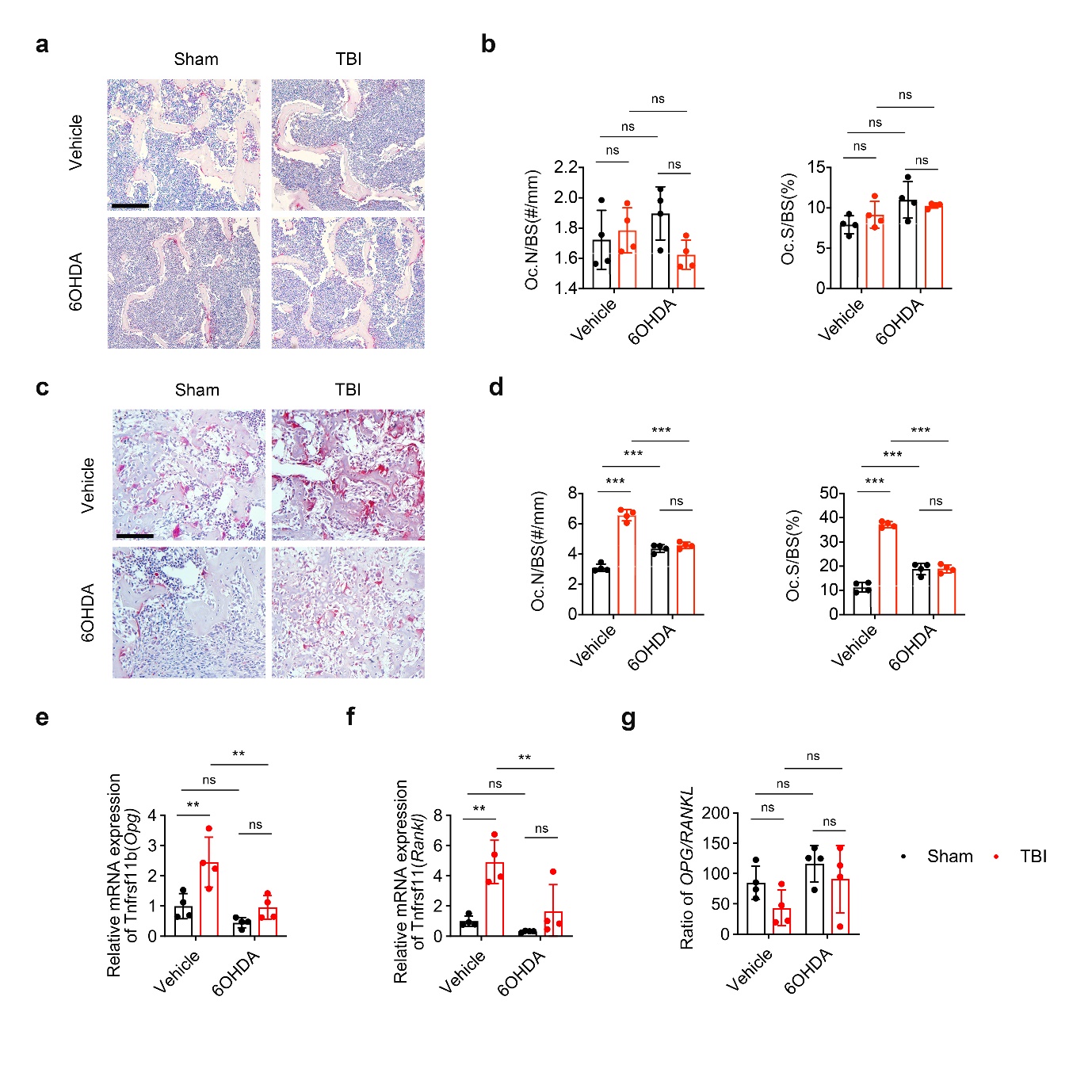


Supplementary Fig. 9 TBI the increased bone turnover rate through sympathetic tone. **a-b** Representative images of TRAP staining and quantitative analysis of osteoclasts in bone marrow from 4-month-old male mice in sham group, 6OHDA group, TBI group, and TBI + 6OHDA group at 14^th^ day post operation. Scale bar: 200 μm. **c-d** Representative images of TRAP staining and quantitative analysis of osteoclasts in callus from 4-month-old male mice in sham group, 6OHDA group, TBI group, and TBI + 6OHDA group at 14^th^ day post operation. Scale bar: 100 μm. **e-g** Quantitative analysis of expression of OPG, RANKL and the OPG/RANKL ratio in callus from 4-month-old male mice in sham group, 6OHDA group, TBI group, and TBI + 6OHDA group at 14^th^ day post operation. All data are presented as means ± standard error of the mean (SEM). **p* < 0.05, ***p* < 0.01 and ****p* < 0.001, ns: not significant. Statistical significance was determined by two-way ANOVA.

Figure. S10.


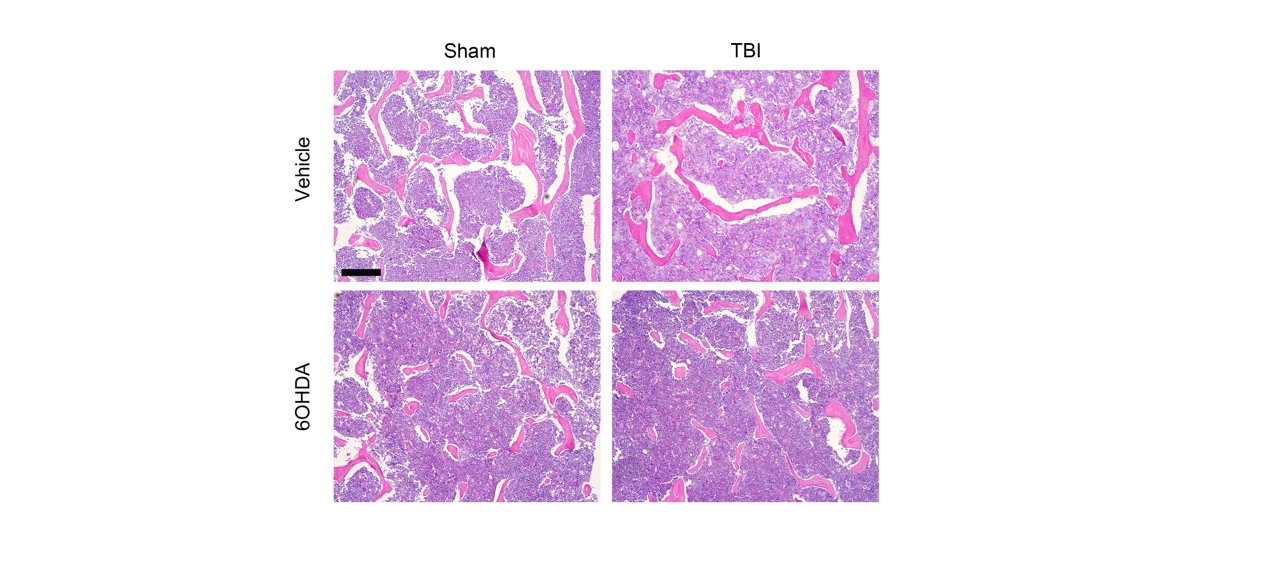


Supplementary Fig. 10 Representative images of H&E staining of callus from 4-month-old male mice in sham group, 6OHDA group, TBI group, and TBI + 6OHDA group at 14^th^ day post operation. Scale bar: 200 μm.

Figure. S11.


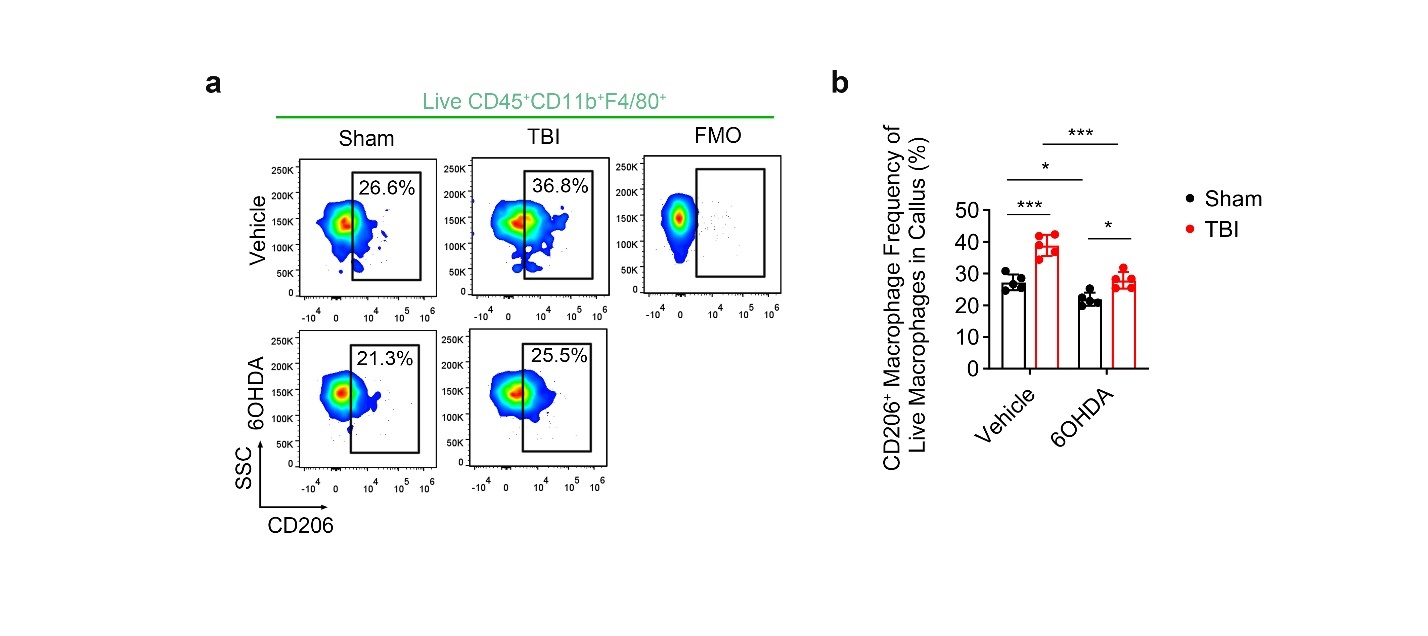


Supplementary Fig. 11 **a**, **b** Representative images of flow cytometry and quantitative analysis of M2 macrophages isolated from callus of 4-month-old male mice in sham group, 6OHDA group, TBI group, and TBI + 6OHDA group at 14^th^ day post operation. All data are presented as means ± standard error of the mean (SEM). **p* < 0.05, ***p* < 0.01 and ****p* < 0.001, ns: not significant. Statistical significance was determined by two-way ANOVA.

Figure. S12.


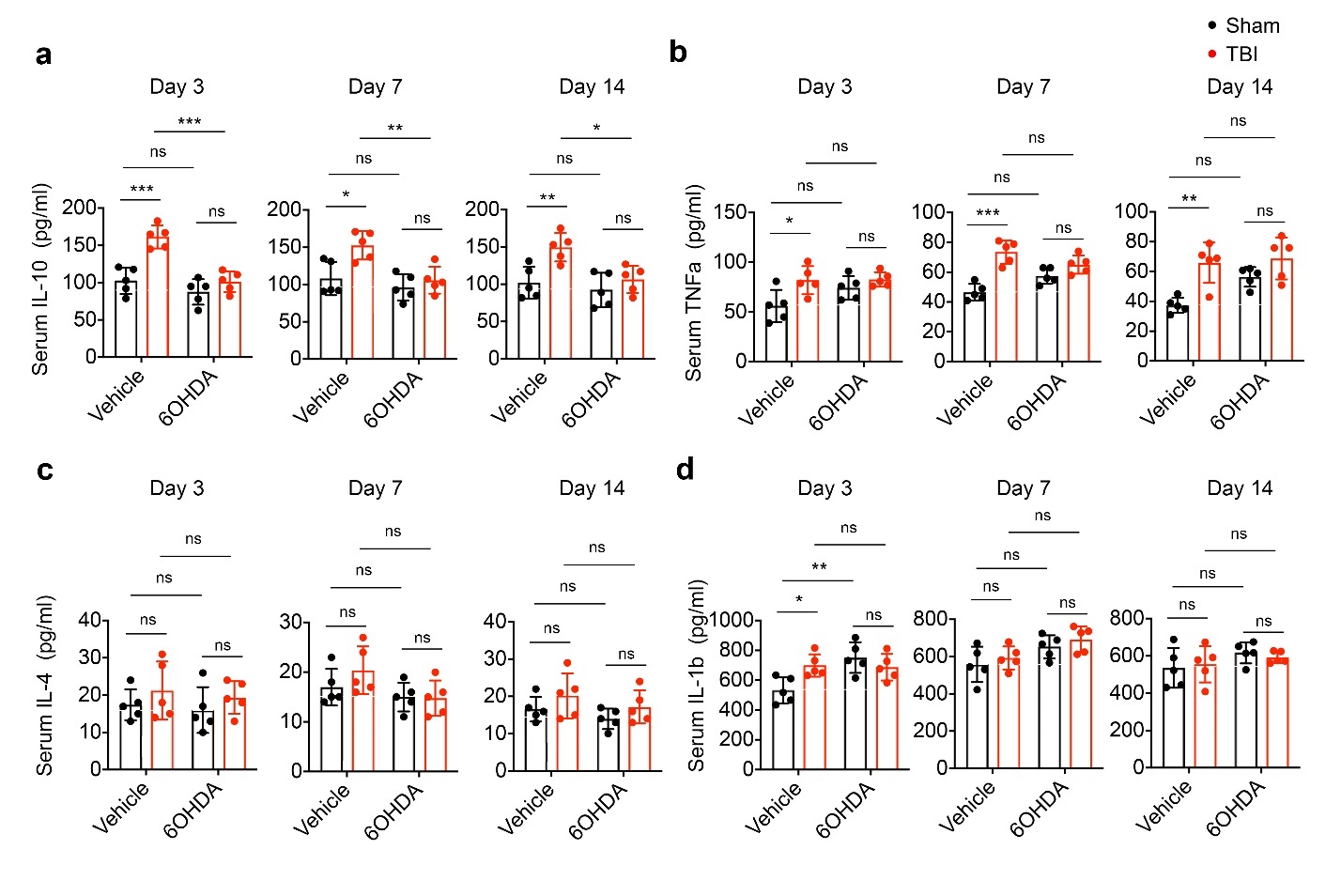


Supplementary Fig. 12 **a**-**d** Quantitative analysis of serum IL-1b, TNFa, IL-4 and IL-10 by ELISA assay from 4-month-old male mice in sham group, 6OHDA group, TBI group, and TBI + 6OHDA group at third, 7^th^ and 14^th^ day post operation. All data are presented as means ± standard error of the mean (SEM). **p* < 0.05, ***p* < 0.01 and ****p* < 0.001, ns: not significant. Statistical significance was determined by two-way ANOVA.

Figure. S13.


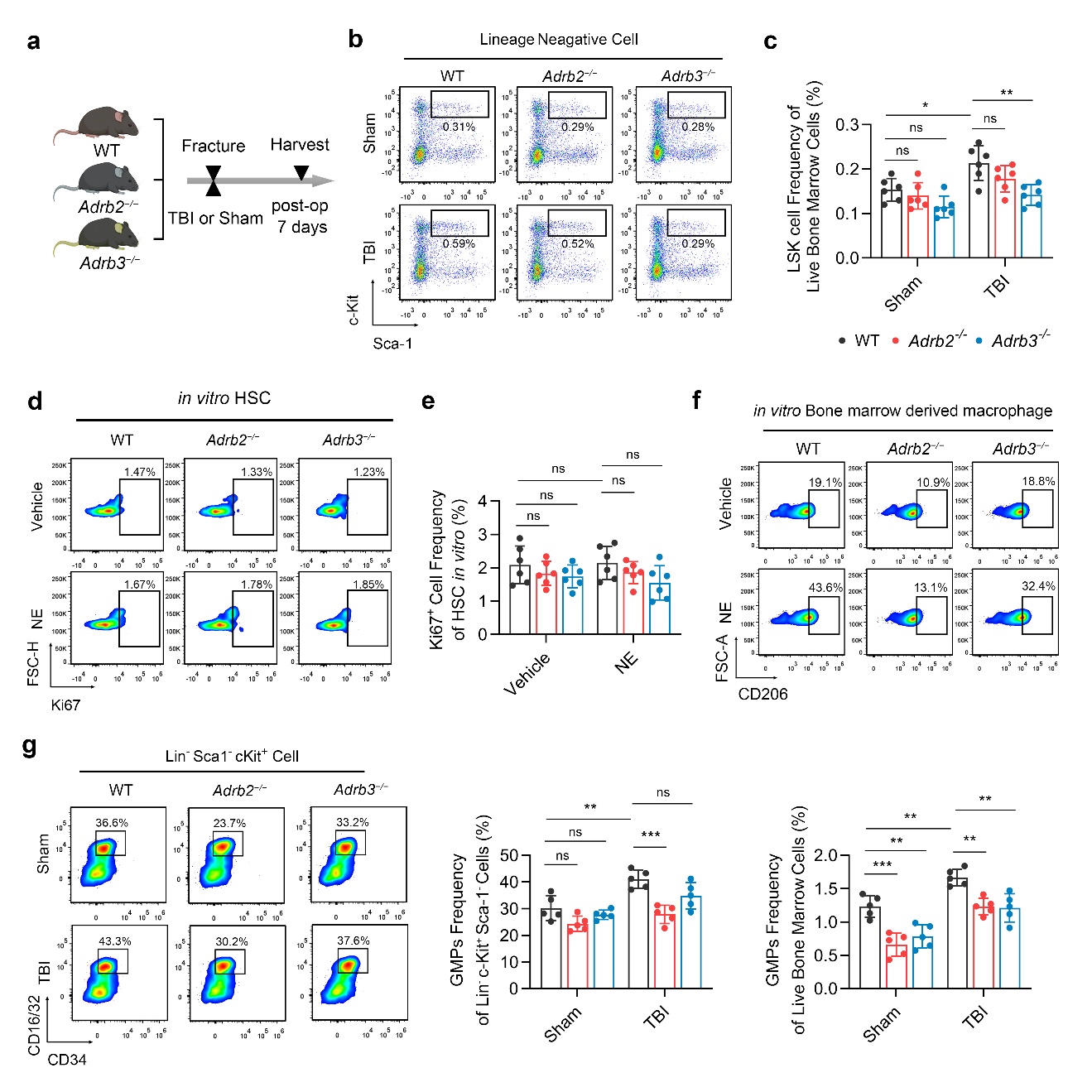


Supplementary Fig. 13 **a** Schematic graph of the investigating the role of *Adrb2* and *Adrb3* in hematopoiesis. **b, c** Representative flow cytometry plots and quantitative analysis of Lin^−^ Sca-1^+^ c-Kit^+^ cells (LSKs) isolated from 4-month-old male fractured mice in sham group and TBI group from WT, *Adrb2*^−/−^, and *Adrb3*^−/−^ mice. **d, e** Representative flow cytometry plots and quantitative analysis of Ki67^+^ hematopoietic stem cells cultured in vitro. **f** Representative flow cytometry plots of CD206^+^ bone marrow derived macrophages isolated from WT, *Adrb2*^−/−^, and *Adrb3*^−/−^ mice. **g** Representative flow cytometry plots and quantitative analysis of Lin^−^ Sca-1^−^ c-Kit^+^ CD34^+^ CD16/32^+^ cells (GMPs) isolated from 4-month-old male fractured mice in sham group and TBI group from WT, *Adrb2*^−/−^, and *Adrb3*^−/−^ mice. All data are presented as means ± standard error of the mean (SEM). **p* < 0.05, ***p* < 0.01 and ****p* < 0.001, ns: not significant. Statistical significance was determined by two-way ANOVA.

Table S1.

Supplementary Table 1 Information of Patients

| Number | Gender | Age | TBI | GCS Score | Side of Fracture and Injured Hemisphere | Union at Sixth month | Basic Disease |
| --- | --- | --- | --- | --- | --- | --- | --- |
| 1 | Male | 30 | No | 15 | - | No |  |
| 2 | Male | 51 | No | 15 | - | No |  |
| 3 | Female | 47 | No | 15 | - | No | Lower leg amputation |
| 4 | Male | 39 | No | 15 | - | No |  |
| 5 | Male | 44 | No | 15 | - | Yes |  |
| 6 | Male | 30 | No | 15 | - | No |  |
| 7 | Female | 67 | No | 15 | - | No | Hypertension |
| 8 | Female | 74 | No | 15 | - | Yes |  |
| 9 | Male | 25 | No | 15 | - | Yes |  |
| 10 | Male | 60 | No | 15 | - | Yes | Hypertension |
| 11 | Male | 18 | No | 15 | - | No |  |
| 12 | Male | 45 | No | 15 | - | No |  |
| 13 | Male | 39 | No | 15 | - | No |  |
| 14 | Male | 37 | No | 15 | - | Yes |  |
| 15 | Female | 72 | No | 15 | - | No |  |
| 16 | Male | 42 | No | 15 | - | Yes |  |
| 17 | Male | 42 | No | 15 | - | Yes |  |
| 18 | Male | 31 | No | 15 | - | No |  |
| 19 | Male | 33 | No | 15 | - | Yes |  |
| 20 | Male | 41 | No | 15 | - | No |  |
| 21 | Female | 43 | No | 15 | - | No |  |
| 22 | Male | 62 | Yes | 9 | Contralateral | Yes |  |
| 23 | Male | 50 | Yes | 11 | Contralateral | Yes | Hypertension |
| 24 | Male | 39 | Yes | 13 | Ipsilateral | Yes |  |
| 25 | Male | 68 | Yes | 12 | Contralateral | No | Deep venous thrombosis |
| 26 | Female | 56 | Yes | 8 | Ipsilateral | Yes |  |
| 27 | Male | 18 | Yes | 10 | Ipsilateral | Yes |  |
| 28 | Male | 57 | Yes | 13 | Contralateral | No |  |
| 29 | Male | 33 | Yes | 14 | Contralateral | No |  |
| 30 | Male | 31 | Yes | 12 | Ipsilateral | Yes |  |
| 31 | Female | 47 | Yes | 13 | Contralateral | Yes |  |
| 32 | Male | 19 | Yes | 11 | Contralateral | Yes |  |
| 33 | Male | 18 | Yes | 8 | Ipsilateral | Yes |  |
| 34 | Male | 22 | Yes | 12 | Contralateral | No |  |
| 35 | Female | 61 | Yes | 10 | Contralateral | Yes | Hypertension |
| 36 | Male | 55 | Yes | 13 | Contralateral | No |  |
| 37 | Female | 86 | Yes | 12 | Ipsilateral | No | Aortic atherosclerosis |
| 38 | Male | 49 | Yes | 9 | Contralateral | Yes |  |
| 39 | Female | 54 | Yes | 11 | Contralateral | Yes |  |
| 40 | Male | 40 | Yes | 10 | Ipsilateral | Yes |  |
| 41 | Male | 34 | Yes | 12 | Contralateral | Yes |  |

GCS score, Glasgow coma scale score.

Table S2.

Supplementary Table 2 Clinical information of patients

| Variables | Fracture | Fracture and TBI | P value |
| --- | --- | --- | --- |
| Age, Mean (SD) | 43.44 (14.76) | 44.95 (18.40) | 0.7573 |
| Sex |  |  | > 0.99 |
| Male | 16 | 15 |  |
| Female | 5 | 5 |  |
| Fracture healing |  |  | 0.0278 |
| Union at 6th month | 8 | 15 |  |
| Nonunion at 6th month | 13 | 5 |  |
